# Meta-analysis of transcriptome data identifies a novel 5-gene pancreatic adenocarcinoma classifier

**DOI:** 10.18632/oncotarget.8139

**Published:** 2016-03-16

**Authors:** Manoj K. Bhasin, Kenneth Ndebele, Octavian Bucur, Eric U. Yee, Hasan H. Otu, Jessica Plati, Andrea Bullock, Xuesong Gu, Eduardo Castan, Peng Zhang, Robert Najarian, Maria S. Muraru, Rebecca Miksad, Roya Khosravi-Far, Towia A. Libermann

**Affiliations:** ^1^ Department of Medicine, BIDMC Genomics, Proteomics, Bioinformatics and Systems Biology Center, Harvard Medical School and Beth Israel Deaconess Medical Center, Boston, MA, USA; ^2^ Department of Pathology, Harvard Medical School and Beth Israel Deaconess Medical Center, Boston, MA, USA; ^3^ Department of Molecular Cell Biology, Institute of Biochemistry of the Romanian Academy, Bucharest, Romania; ^4^ Department of Electrical and Computer Engineering, University of Nebraska-Lincoln, Lincoln, Nebraska, USA; ^5^ Division of Hematology and Oncology, Harvard Medical School and Beth Israel Deaconess Medical Center, Boston, MA, USA

**Keywords:** pancreatic cancer, biomarkers, transcriptome, bioinformatics, meta-analysis

## Abstract

**Purpose:**

Pancreatic ductal adenocarcinoma (PDAC) is largely incurable due to late diagnosis. Superior early detection biomarkers are critical to improving PDAC survival and risk stratification.

**Experimental Design:**

Optimized meta-analysis of PDAC transcriptome datasets identified and validated key PDAC biomarkers. PDAC-specific expression of a 5-gene biomarker panel was measured by qRT-PCR in microdissected patient-derived FFPE tissues. Cell-based assays assessed impact of two of these biomarkers, TMPRSS4 and ECT2, on PDAC cells.

**Results:**

A 5-gene PDAC classifier (TMPRSS4, AHNAK2, POSTN, ECT2, SERPINB5) achieved on average 95% sensitivity and 89% specificity in discriminating PDAC from non-tumor samples in four training sets and similar performance (sensitivity = 94%, specificity = 89.6%) in five independent validation datasets. This classifier accurately discriminated PDAC from chronic pancreatitis (AUC = 0.83), other cancers (AUC = 0.89), and non-tumor from PDAC precursors (AUC = 0.92) in three independent datasets. Importantly, the classifier distinguished PanIN from healthy pancreas in the PDX1-Cre;LSL-Kras^G12D^ PDAC mouse model. Discriminatory expression of the PDAC classifier genes was confirmed in microdissected FFPE samples of PDAC and matched surrounding non-tumor pancreas or pancreatitis. Notably, knock-down of TMPRSS4 and ECT2 reduced PDAC soft agar growth and cell viability and TMPRSS4 knockdown also blocked PDAC migration and invasion.

**Conclusions:**

This study identified and validated a highly accurate 5-gene PDAC classifier for discriminating PDAC and early precursor lesions from non-malignant tissue that may facilitate early diagnosis and risk stratification upon validation in prospective clinical trials. Cell-based experiments of two overexpressed proteins encoded by the panel, TMPRSS4 and ECT2, suggest a causal link to PDAC development and progression, confirming them as potential therapeutic targets.

## INTRODUCTION

Pancreatic ductal adenocarcinoma (PDAC), the third leading cause of cancer death in the United States (US), is marked by an exceptionally high mortality rate [1], due to late diagnosis when curative resection is no longer possible. Although imaging and endoscopic approaches assist with PDAC staging, their efficacy is limited for screening and risk stratification, and PDAC diagnosis can be limited by indeterminate pathologic evaluation of biopsy specimens [2]. Therefore, superior biomarkers for earlier detection of PDAC and for improved risk stratification are imperative for improving PDAC survival.

The magnitude of the need for better PDAC biomarkers is large: 330,000 patients worldwide die from PDAC annually and many must face uncertainty of diagnostic tests or the malignant potential of incidentally discovered pancreatic lesions and PDAC risk factors. For example, the limits of cytologic examination of pancreatic mass lesions often preclude definitive diagnosis of PDAC, particularly in the presence of chronic pancreatitis and when an on-site cytopathologist is not available [3, 4]. Moreover, rapid improvements in imaging quality and the number of imaging procedures (26 million annually in the US) have led to a rise in identification of potential PDAC precursor lesions such as intraductal papillary mucinous neoplasms (IPMNs) and mucinous cystic neoplasms (MCNs). Although resection of precursor lesions is associated with better survival, it is often uncertain which lesions will progress to invasive cancer and morbidity and mortality of surgery can be high [5]. Accurate biomarkers to aid risk stratification would greatly improve the current diagnostic and decision-making quandary for these patients. Similarly, accurate biomarkers are greatly needed to improve screening, particularly for those who may be at increased risk of developing PDAC: family history of PDAC, hereditary syndromes, chronic pancreatitis, type 3c diabetes, smokers, BRCA2 carriers, etc. [6].

Several serum-based (CA19-9, CA125) and tissue-specific (macrophage inhibitory cytokine-1, *K-ras*, mesothelin, PSCA, mucins, SMAD4, p53 mutations) proteins have been tested as potential PDAC diagnostic biomarkers. All have failed to demonstrate the accuracy needed for early detection or screening [7]. CA19-9 is used clinically to monitor PDAC response to therapy, but its utility for screening and risk-assessment is limited: it can be elevated in benign intra-abdominal processes and normal when PDAC tumors are small, the time when resolving diagnostic uncertainty is most important [8].

The urgent need for improved PDAC diagnosis has spurred a number of studies aimed at identifying differentially expressed genes in PDAC. However, no transcriptome data has yet translated into a clinically useful biomarker. Integration of the literature on candidate PDAC biomarkers resulted in identification of several thousand differentially expressed PDAC genes [9, 10]. The relevance of these genes for PDAC remains unclear due to inherent statistical limitations of the applied approaches combined with batch effects, variable techniques and platforms, and varying analytic methods [11]. Lack of concordance of published gene signatures of individual microarray studies due to variability in analytical strategies makes comparative analysis difficult when standard approaches are used [11].

One alternative to overcome the limitations of analyzing individual microarray datasets or multiple datasets that have been processed and normalized by different approaches is meta-analysis of multiple transcriptional profiling studies applying identical analytics that can generate gene signatures with increased reproducibility and sensitivity, revealing biological insight not evident in the original datasets [12]. The increased statistical power of this approach may identify a more reliable transcriptome signature by detecting potentially important genes missed in a single study or in an analysis of multiple studies using divergent analytical methods and eliminating false positives [11]. We now report the identification of a 5-gene PDAC classifier based on our meta-analysis of publicly available PDAC microarray datasets that accurately discriminates PDAC and early PDAC precursors from benign pancreatic lesions and healthy controls. We demonstrate validation of these 5 genes as PDAC-specific in FFPE samples from patients with PDAC, providing strong support for the predicted diagnostic performance of our 5-gene PDAC classifier.

## RESULTS

### Dataset identification and meta-analysis strategy

**Figure 1:**
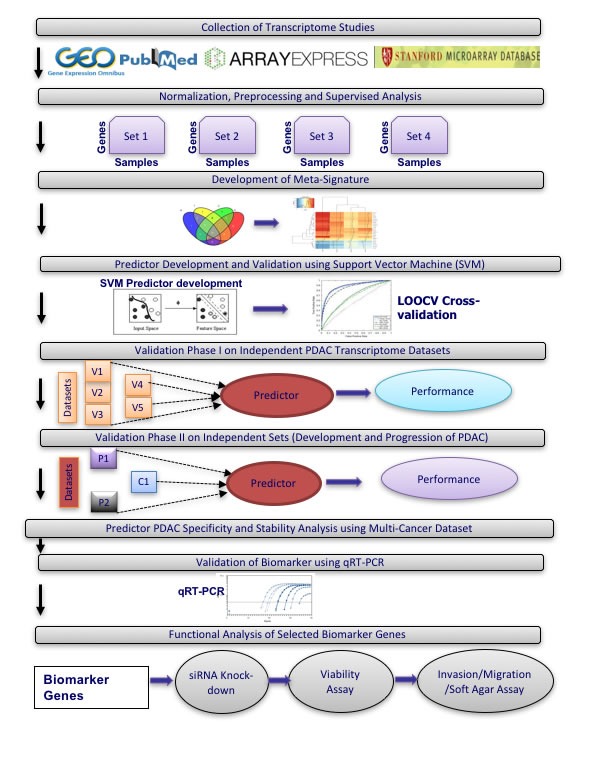
Overview of meta-analysis approach for development and validation of PDAC biomarker biomarker panel

To identify biomarkers that accurately discriminate between PDAC and normal pancreas or benign pancreatic lesions, we selected publicly available transcriptional profiling datasets for meta-analysis. These datasets were divided into training sets for development of a PDAC biomarker classifier and independent validation sets (see overview of meta-analysis strategy in Figure 1).

Among the publicly available microarray repositories, we selected four transcriptional profiling datasets of normal pancreas and PDAC tissue samples as training sets (Figure 1 and Table 1A), including two datasets from microdissected pancreatic tissue (GSE18670: 6 normal, 6 PDAC; E-MEXP-950: 9 normal, 13 PDAC) and two datasets from whole tissue (GSE15471: 35 normal, 35 PDAC; GSE16515: 14 normal, 36 PDAC) (Table 1A). In Phase I of independent validation, performance of the optimized PDAC biomarker panel was validated on five additional datasets (Table 1B). Phase I Validation was restricted to Affymetrix datasets from clinical samples similar to the training sets, namely comparison of normal versus PDAC samples (two datasets: GSE32676: 7 normal, 25 PDAC; GSE28735: 45 normal, 45 PDAC), only PDAC samples (2 datasets: GSE9599: 36 PDAC; E-MEXP-2894: 18 PDAC) and only normal pancreas tissue (1 dataset: E-TABM-145: 145 normal). In Phase II Validation, PDAC biomarker panel performance was tested on four additional independent datasets that compared results from: i) PDAC versus normal pancreatic tissue on Agilent microarrays (GSE11838: 4 normal, 28 PDAC), ii) PDAC versus other cancers (breast, colon, liver, lung, prostate) (GSE12630: 25 other cancers, 11 PDAC), iii) PDAC versus chronic pancreatitis (E-MEXP-1121: 9 pancreatitis, 6 PDAC), and iv) normal pancreas versus PDAC precursor lesions (IPMN with low- to intermediate-grade dysplasia (LIGD-IPMN, previously called intraductal papillary-mucinous adenoma (IPMA)), IPMN with high-grade dysplasia (HGD-IPMN, previously called intraductal papillary-mucinous carcinoma (IPMC)) and IPMN with associated invasive carcinoma (InvCa-IPMN, previously called invasive cancer originating from IPMN) (GSE19650: 6 normal, 15 PDAC precursors (5 LIGD-IPMN, 5 HGD-IPMN, 5 InvCa-IPMN)) (Figure 1 and Table 1B). All datasets utilized oligonucleotide-based microarray platforms (three versions of Affymetrix GeneChips and Agilent microarrays in one dataset).

**Table 1A:** Datasets used for development of PDAC classifier

Training Sets:					
Dataset	Normal	Tumor	Sample Type	Platform	Accession #
Set1	6	6	Enriched	U133 Plus 2.0	GSE18670
Set2	9	13	Microdissected	U133A	E-MEXP-950
Set3	35	35	Whole Tissue	Plus 2.0	GSE15471
Set4	14	36	Whole Tissue	Plus 2.0	GSE16515

Number of samples reflects only the samples that were used for the meta-analysis, after removing samples with low quality, outlier arrays.

**Table 1B:** Datasets used for independent validation of PDAC classifier

Phase I Validation Sets:					
Dataset	Normal	Tumor	Sample Type	Platform	Accession #
V1	7	25	Whole Tissue	Plus 2.0	GSE32676
V2	45	45	Whole Tissue	Gene St 1.0	GSE28735
V3	0	36	Whole Tissue (Xenografts in duplicate)	Plus 2.0	GSE9599
V4	0	18	Microdissected	Plus 2.0	E-MEXP-2894
V5	145	0	Whole Tissue & Cell Lines (most 3 replicates)	U133A	E-TABM-145
**Phase II Validation Sets:**					
**Dataset**	**Group**	**Pancreatic Tumor**	**Sample Type**	**Platform**	**Accession #**
P1	6 (Normal)	15 (IPMA, IPMC, IPMN)	Microdissected	Plus 2.0	GSE19650
P2	9 (Pancreatitis)	6	Microdissected	U133A	E-MEXP-1121
C1	4 (Normal)	28	Whole Tissue	Agilent	GSE11838
M1	25 (other Cancers)	11	Whole Tissue	U133A	GSE12630

### Identification of PDAC biomarker candidates

Using identical normalization and statistical methods for each dataset, a broad range of differentially expressed genes was identified through empirical Bayes comparative meta-analysis of the raw expression data in the four PDAC training datasets. The number of differentially expressed genes ranged from 90 to 10,169 genes (See Supplementary Table S1), totaling 11,322 significantly differentially expressed genes in the four training datasets. Heatmaps for the top up- and down-regulated genes in two of the datasets are shown in Supplementary Figure S1A. Venn diagram analysis of these differentially expressed genes identified 409 genes with concordant directionality to at least three of the four datasets (Supplementary Figure S1B). These 409 genes were selected for further evaluation.

**Figure 2:**
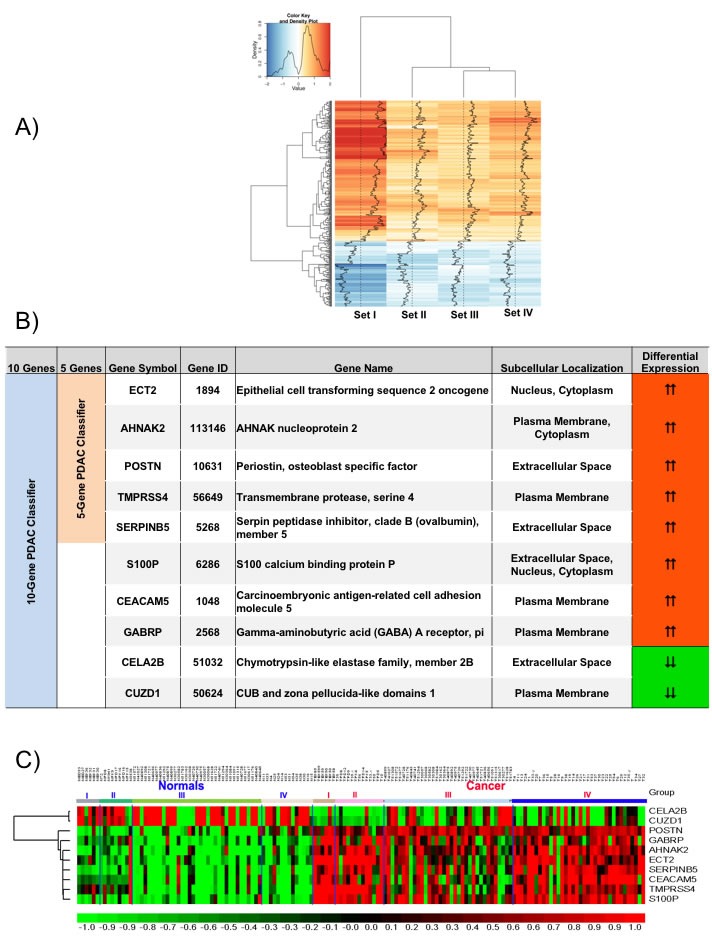
Meta-signature of genes that are consistently differentially expressed in multiple datasets and candidate PDAC diagnostic biomarker panels **A.** Signal to Noise ratio based heatmap of the 409 meta-signature genes. **B.** Description of the genes from the 5- and 10-gene based PDAC biomarker panels. **C.** Relative expression of the 10 candidate biomarker genes across all four training sets visualized as a heatmap.

Consistent expression across these four datasets for each of the 409 concordant genes is demonstrated in a heatmap of the relative ratio of gene expression in PDAC compared to normal pancreas (NP) (Figure 2A), with the extent of overexpression or underexpression denoted by red or blue shading, respectively. Principal Component Analysis (PCA) of these 409 genes shows a dominant separation pattern for most of the PDAC and normal pancreas samples in each dataset (Supplementary Figure S1C).

Canonical pathway analysis of these 409 common PDAC genes using Ingenuity Pathway Analysis identified the highest statistical enrichment of these genes in various cancer-related pathways linked to DNA damage (ATM Signaling, DNA damage-induced 14-3-3σ Signaling, GADD45 signaling), cell cycle (G2/M DNA damage checkpoint regulation, Cell cycle control of chromosomal replication, p53 signaling, Mitotic role of Polo-like kinase), and Protein kinase A signaling (Supplementary Figure S2). Most of these pathways play important roles in PDAC.

### Class prediction analysis in training sets

Class prediction analysis with support vector machines generated a large number of PDAC classifiers containing 2 to 40 genes. Based on LOOCV evaluation in the training sets, classifiers containing 5 or 10 genes performed with highest accuracy. The 5-gene PDAC classifier (TMPRSS4, ECT2, SERPINB5, AHNAK2, POSTN) is a subset of the 10-gene PDAC classifier (TMPRSS4, ECT2, SERPINB5, AHNAK2, POSTN, S100P, CEACAM5, GABRP, CELA2B, CUZD1) and only includes genes overexpressed in PDAC (Figure 2B). The 10-gene PDAC classifier includes 2 genes (CELA2B, CUZD1) that are reduced specifically in PDAC. A heatmap of these 10 genes across the four training sets (Figure 2C) demonstrates differential expression in PDAC compared to normal pancreas across most of the samples.

**Figure 3:**
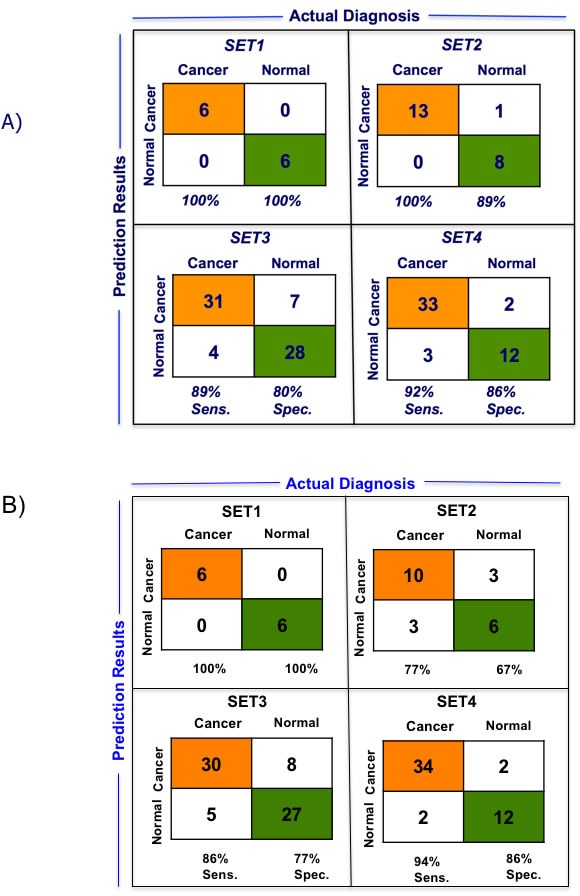

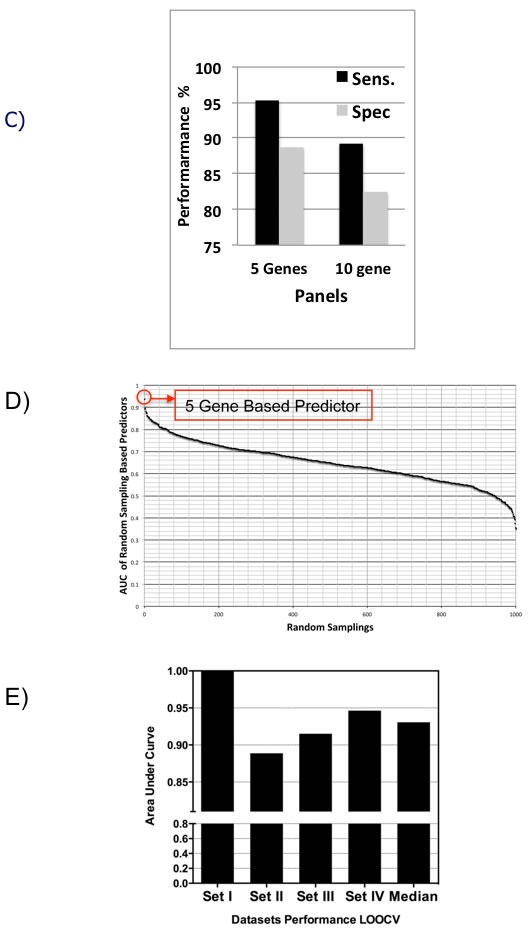
Performance of 5-gene and 10-gene PDAC Classifiers on training sets using leave one out cross-validation (LOOCV) **A.** Diagnostic performance of the 5-gene PDAC classifier on the four training sets of PDAC vs. normal pancreas. Sensitivity (Sens.) and specificity (Spec.) are indicated below each set. **B.** Diagnostic performance of the 10-gene PDAC classifier on the four training sets of PDAC vs. normal pancreas. Sensitivity (Sens.) and specificity (Spec.) are indicated below each set. **C.** Comparison of the performance of the 5- and 10-gene PDAC classifiers across the four training sets. **D.** AUC curve for random sampling based prediction. The ranking of the 5-gene PDAC classifier is indicated. **E.** AUC plot for 5-gene PDAC classifier across the four training sets and for the median of the four datasets.

We performed LOOCV of the 10-gene and 5-gene PDAC classifier across the four training datasets in order to compare predictive performance and to determine which of these two classifiers to further evaluate on the independent test sets. LOOCV of the 5-gene classifier demonstrated overall better performance than the 10-gene predictor for the four training sets. While for each of the four training datasets individually sensitivity ranges from 0.89-1.0 and specificity from 0.80-1.00 for the 5-gene predictor (Figure 3A), for the 10-gene predictor sensitivity ranges from 0.77-1.0 and specificity from 0.67-1.00 (Figure 3B). Comparison of the 5- and 10-gene PDAC classifier performance shows an average 0.95 sensitivity and 0.89 specificity for the 5-gene classifier, in contrast to 0.89 sensitivity and 0.83 specificity for the 10-gene classifier (Figure 3C). Based on this comparison of the 10-gene PDAC classifier to the 5-gene classifier, only the 5-gene classifier was further evaluated on the independent datasets. Random sampling based ROC prediction across the training sets further confirms that the 5-gene PDAC predictor has indeed the largest AUC (Figure 3D): AUC for the four datasets ranged from 0.88-1.0 with median=0.93 (Figure 3E, demonstrates threshold independent performance).

Evaluation of the GENT database that compares relative expression of genes between different cancers and their normal tissue counterparts indicates that all genes in the 5-gene classifier are overexpressed in PDAC relative to normal pancreas (Supplementary Figure S3). Each of these 5 genes appears also overexpressed in several other types of cancer (Supplementary Figure S3).

### The 5-gene PDAC classifier predicts PDAC with high accuracy in 9 independent validation sets

**Figure 4:**
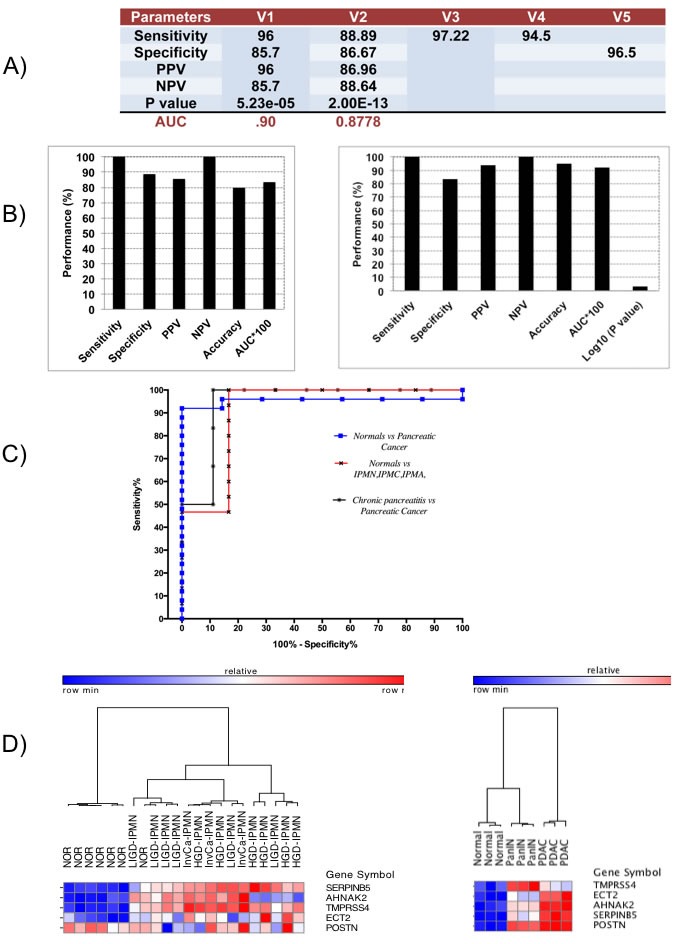
Performance and expression of 5-gene PDAC classifier on independent validation sets **A.** Diagnostic performance of 5-gene PDAC classifier on five independent validation sets of normal and PDAC tissue samples. PPV = positive predictive value, NPV = negative predictive value. **B.** Diagnostic performance of the 5-gene PDAC classifier on Pancreatitis vs. Pancreatic Cancer dataset (Left) and on a dataset of normal pancreatic duct compared to LIGD-IPMN, HGD-IPMN, and InvCa-IPMN (Right). **C.** ROC curves for the different independent validation datasets. **D.** Hierarchical clustering analysis showing performance and expression of the 5-gene PDAC classifier on normal pancreatic duct compared to LIGD-IPMN, HGD-IPMN, and InvCa-IPMN samples. Red = upregulated, Blue = downregulated (Left Heatmap). Cross-species performance of the 5-gene PDAC classifier on a GEM mouse model of PDAC (Right Heatmap). Hierarchical clustering of pancreatic tissue samples from three mice each for normal pancreas, PanIN and PDAC across the 5-gene PDAC panel. Red = upregulated, Blue = downregulated.

In Phase I Validation, the 5-gene PDAC classifier accurately predicted the class of PDAC compared to NP with a sensitivity of 96% and 88.89%, a specificity of 85.7 and 86.67%, and AUC of 0.9 and 0.8778 in two independent validation sets that contained 25 and 45 PDAC and 7 and 45 normal pancreas samples, respectively (Figure 4A and Table 1B). These results are significantly better than various published values for CA19-9 [13, 14]. In two datasets containing exclusively PDAC samples a sensitivity of 97.22% and 94.5% was achieved and in a dataset containing 145 normal samples a specificity of 96.5% was determined.

In Phase II Validation, we tested the 5-gene PDAC classifier on a dataset that includes 6 PDAC samples and 9 chronic pancreatitis samples (Table 1B). Specificity was 88.9% and sensitivity 100% with an overall accuracy of 80% and an AUC of 0.83 (Figure 4B, Left Graph). Since discrimination between chronic pancreatitis and PDAC is a key clinical challenge, the fact that the 5-gene PDAC classifier accurately distinguishes between PDAC and pancreatitis is a further important validation step for this 5-gene biomarker panel. We similarly tested the 5-gene PDAC classifier on an independent validation dataset containing laser microdissected normal main pancreatic duct epithelial cells and neoplastic epithelial cells from potential PDAC precursor lesions, LIGD-IPMN, HGD-IPMN and InvCa-IPMN [15]. The 5-gene predictor separated LIGD-IPMN, HGD-IPMN and InvCa-IPMN from normal pancreatic duct epithelial cells with 100% sensitivity and 83% specificity, achieving an AUC of 0.92 (Figure 4B, Right Graph). The ROC curves for normal vs. PDAC, chronic pancreatitis vs. PDAC, and normal vs. LIGD-IPMN, HGD-IPMN, and InvCa-IPMN are shown in Figure 4C.

Hierarchical clustering of this dataset demonstrates that the 5-gene PDAC classifier separates all potential PDAC precursor (LIGD-IPMN, HGD-IPMN, InvCa-IPMN) samples from the normal pancreatic duct samples except for one normal sample (Figure 4D, Left Heatmap). Four classifier genes are consistently overexpressed in the PDAC precursor samples. TMPRSS4 and SERPINB5 are increased relative to 6 of the 7 normal pancreas samples in 100% of the PDAC precursor samples. AHNAK2 is elevated in 87% and ECT2 in 80% of the PDAC precursor samples. POSTN was not differentially expressed, but its expression had little impact on overall performance of the classifier in this dataset.

Applying the 5-gene classifier to a dataset that included 11 PDAC samples and 25 tumor samples of various origins (breast, colon, liver, lung, prostate) resulted in sensitivity of 72.73% and specificity of 96% (Supplementary Figure S4A). The ROC curve is shown in Supplementary Figure S4A. These results suggest that the 5-gene classifier not only discriminates PDAC and its precursors from normal pancreas and benign pancreatic lesions, but also from several other types of cancer.

While all datasets analyzed above were derived on the Affymetrix platform, one small dataset on the Agilent microarray platform was available (GSE11838). Cross-platform evaluation on this dataset of PDAC versus normal pancreas demonstrated a sensitivity and specificity of 96.4% and 75% respectively and an AUC of 0.84 (Supplementary Figure S4B).

### The 5-gene PDAC classifier distinguishes between PDAC or early stage PDAC, PanIN, and healthy pancreas in the PDX1-Cre;LSL-Kras^G12D^ GEM model of PDAC

While IPMNs have the potential to become malignant and progress towards PDAC, the majority of PDAC cases likely evolve from pancreatic intraepithelial neoplasia [PanIN] lesions containing Kras mutations [16]. While PanINs are difficult to detect in humans, various mutant Kras GEM models exist that spontaneously develop PDAC through the stages of PanIN development [17]. One GEM PDAC model is the PDX1-Cre;LSL-Kras^G12D^ model [17]. These mice develop low and high-grade progressive ductal PanIN lesions with increasing age and low frequency progression to invasive PDAC upon activation of oncogenic Kras in the pancreas, phenocopying development of human PDAC [17, 18]. We applied an unsupervised learning approach, hierarchical clustering, to one available Affymetrix microarray dataset of three biological replicates each of normal pancreatic tissue, PanIN and PDAC from the PDX1-Cre;LSL-Kras^G12D^ mice using the expression values of our five PDAC classifier genes [18]. Hierarchical clustering of this dataset using the equivalent mouse GeneIDs demonstrated that all 5 genes were upregulated in both PanINs and PDAC compared to normal pancreas, resulting in perfect separation of PanINs and PDAC samples from normal pancreas (Figure 4D, Right Heatmap). Interestingly, PanINs perfectly separated from PDAC. PanIN samples clustered on the same main branch as the normal pancreas, but on a separate subbranch within this tree, suggesting that PanIN is indeed a stage different from normal, but in between normal and PDAC. POSTN exhibited the same level of overexpression in PanIN and PDAC compared to normal pancreas, TMPRSS4 was higher expressed in PanIN than PDAC, and ECT2, AHNAK2, and SERPINB5 were higher expressed in PDAC (Figure 4D, Right Heatmap). These results provide the strongest evidence that the 5-gene PDAC classifier is able to discriminate early PDAC precursor lesions from normal pancreas and that differential expression of these 5 genes may even differentiate between PanIN and PDAC, suggesting dynamic, malignancy-related changes of these 5 genes during PDAC development.

### qRT-PCR validation of the 5-gene PDAC classifier in retrospective FFPE patient samples demonstrates overexpression of the 5 genes in PDAC as compared to pancreatitis or healthy pancreas

**Figure 5:**
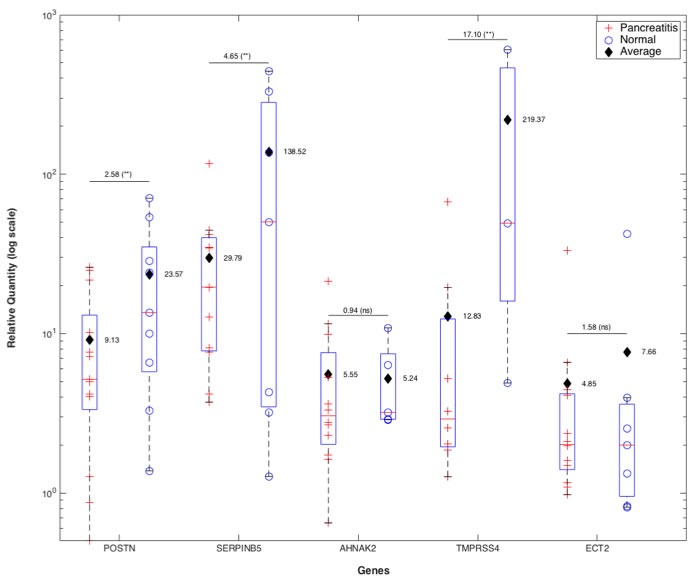
qRT-PCR validation of 5-gene PDAC classifier on retrospective microdissected FFPE samples from patients with PDAC Total RNA was isolated from 22 matched pairs of PDAC and pancreatitis (*n* = 13) or healthy pancreas (*n* = 9). FFPE tissue blocks were inspected by the pathologist and marked regions of PDAC, pancreatitis and healthy pancreas microdissected. QRTPCR was performed on each sample for POSTN, SERPINB5, AHNAK2, TMPRSS4 and ECT2 in duplicates. Box plots of fold change (RQ values) of PDAC samples relative to their matched pancreatitis or normal pancreas samples after normalization to GAPDH are shown. Average fold change values for each gene when the baseline was pancreatitis or normal tissue are indicated separately for each box plot. For each gene the ratio of the average fold change values for PDAC *vs*. pancreatitis or normal pancreas when the baseline was normal *vs*. pancreatitis tissue is indicated above the lines along with the statistical significance of the fold change value distributions in PDAC *vs*. normal or pancreatitis (**:*p* < 0.05, ns:non-significant).

To determine whether the five PDAC classifier genes indeed are higher expressed in PDAC than normal pancreas or benign pancreatic lesions, we developed a qRT-PCR assay for the five genes and evaluated the expression pattern of the 5-gene PDAC classifier in 22 microdissected paired retrospective FFPE patient samples containing PDAC and matched non-tumor normal pancreatic tissue (9 samples) or matched pancreatitis tissue (13 samples) (Figure 5). Relative quantity (RQ) values were calculated by using the matched normal or pancreatitis tissue as the baseline to reflect the fold change in tumor samples. In all 22 matched pairs at least 4 of the 5 genes were elevated in pancreatic tumor tissue as compared to normal or pancreatitis tissue, and the box plots reflecting the relative expression of each gene compared to either matched normal or pancreatitis demonstrate clear discrimination, providing strong support that these 5 genes are selectively overexpressed in PDAC (Figure 5). Most importantly, this differential expression of the 5 genes was validated in PDAC compared to pancreatitis, a clinically highly relevant differential diagnosis. All five genes showed elevated average expression in PDAC tissue compared to normal pancreas or pancreatitis tissue. The degree of upregulation when compared to normal tissue was significantly higher in three (POSTN, SERPINB5, TMPRSS4) of five genes than compared to pancreatitis, suggesting that POSTN, SERPINB5 and TMPRSS4 expression increase gradually from healthy pancreas to pancreatitis to PDAC. AHNAK2 and ECT2 also showed elevated levels of expression in the matched PDAC tissue compared to normal or pancreatitis (fold change ~5-8), but the levels of these fold changes relative to normal and pancreatitis tissues were not significantly different.

### TMPRSS4 knockdown decreases PDAC cell migration, invasion, and anchorage-independent growth

**Figure 6:**
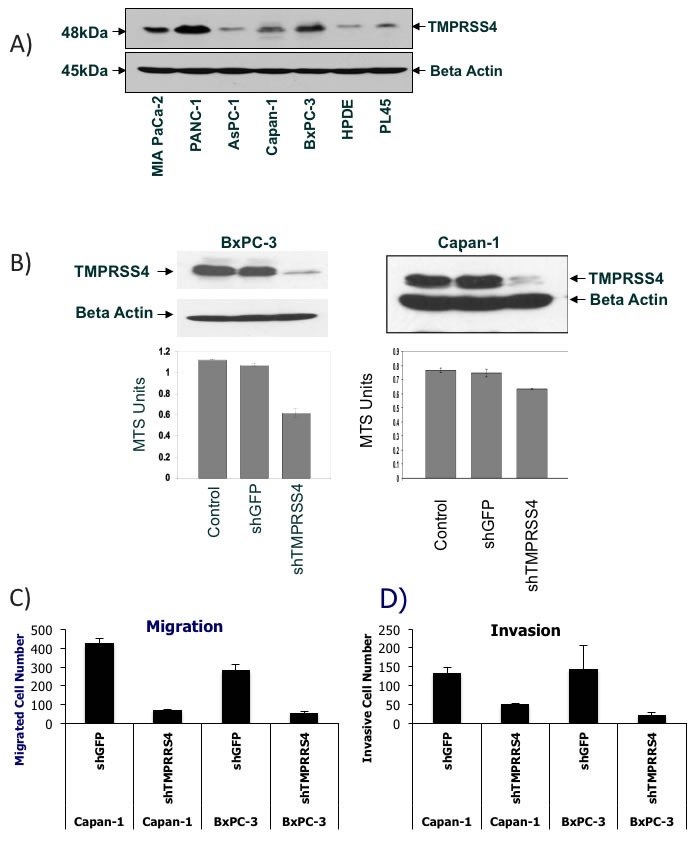
Verification of TMPRSS4 function in various PDAC cells **A.** TMPRSS4 protein expression across various PDAC cell lines and HPDE cells. Western Blot analysis of TMPRSS4 and beta actin. **B.** Cell viability of TMPRSS4 knockdown cells. Western Blot analysis of TMPRSS4 in whole cell lysates from BxPC-3 and Capan-1 cells 72 hours after shTMPRSS4 or shGFP infection (Top). Cell viability analysis of parental cells, shTMPRSS4 and shGFP cells using MTS assay (Bottom). **C.** + **D.** Knockdown of TMPRSS4 reduces migration and invasion of PDAC cells. Capan-1 cells or BxPC-3 cells stably transfected with shGFP-treated cells (control shRNA) or TMPRSS4 shRNA were placed in serum-free culture media and added into the upper compartment of a migration or invasion chamber. After 16 hours, cells in the upper chamber were removed and cells that had migrated or invaded through the pores of the membrane to the other side were fixed, stained, and counted. Cells in five different areas were quantified for migration and invasion studies. C, quantification of cells migrating through fibronectin-coated membranes. D, quantification of cells invading through Matrigel-coated membranes after 16-h incubation and 10% serum as chemoattractant.

To evaluate whether the PDAC classifier encodes proteins that are causally related to PDAC development and progression, we selected two of the genes, TMPRSS4, that has been previously linked to PDAC and other types of cancer [19–25] and ECT2, an oncogene that has previously been shown to be overexpressed in PDAC and other types of cancer [26]. TMPRSS4 protein expression in PDAC has not previously been studied. Western blot analysis verified increased TMPRSS4 protein expression in several PDAC cell lines compared to an immortalized pancreatic epithelial cell line, HPDE (Figure 6A). Particularly high TMPRSS4 expression was observed in MIA PaCa-2, Panc-1, Capan-1 and BxPC-3 PDAC cells. The functional relevance of TMPRSS4 or ECT2 in PDAC cells has not previously been determined, although TMPRSS4 has been demonstrated to induce invasion and epithelial-to-mesenchymal transition ( EMT) of colorectal cancer cells [24, 25] and ECT2 overexpression has been primarily linked to cell proliferation, invasion and migration in lung cancer or glioma cells [26]. We infected two PDAC cell lines (Capan-1, BxPC-3) expressing high levels of TMPRSS4 with lentiviral vectors expressing shRNAs against TMPRSS4 (shTMPRSS4) or lentivirus expressing shGFP and Capan-1 cells with lentiviral vectors expressing shECT2 or scrambled shRNA. ShTMPRSS4 knocked down TMPRSS4 protein expression by more than 80% in both cell lines without affecting β-actin expression (Figure 6B). TMPRSS4 knock-down decreased cell viability, as measured with the MTS assay, in Capan-1 and BxPC-3 cells (Figure 6B). Similar to TMPRSS4, ECT2 knock-down significantly decreased cell viability in Capan-1 cells (Supplementary Figure S6A).

TMPRSS4 knockdown reduced serum-induced migration of Capan-1 and BxPC-3 cells through the pores in a Transwell chamber assay by more than 80% compared with shGFP cells (Figure 6C). Because cell migration promotes tumor invasion, we tested the effect of TMPRSS4 knockdown on cell invasion using Matrigel-coated Transwell chambers. TMPRSS4 knockdown compared with shGFP decreased Capan-1 and BxPC-3 invasion by 60-90% after 16-h incubation (Figure 6D). In contrast, knockdown of ECT2 did not have any significant effect on migration or invasion of Capan-1 cells (Supplementary Figure S6C and D). These results demonstrate that TMPRSS4, but not ECT2 is an inducer of PDAC cell migration and invasion.

One of the hallmarks of malignancy is loss of anchorage-dependent growth as demonstrated by the ability to form colonies in soft agar. TMPRSS4 and ECT2 knockdown in PDAC cells significantly reduced anchorage-independent growth compared with the shGFP and parental cells in soft agar assays (Supplementary Figures S5 and S6B). These results suggest that the 5-gene PDAC classifier includes genes that are directly implicated in pathophysiological mechanisms of PDAC.

## DISCUSSION

We applied an innovative data mining approach to multiple transcriptome datasets followed by class prediction analysis and validation in independent datasets to discover candidate PDAC biomarkers [9, 27]. We identified a 10-gene and, as the most parsimonious and best performing panel and a subset of the 10-gene classifier, a 5-gene PDAC classifier that differentially diagnoses PDAC and precursor lesions with high accuracy. This 5-gene PDAC classifier was validated in 9 independent human datasets, one dataset derived from a GEM mouse model of PDAC and by retrospective qRT-PCR analysis of patient-derived FFPE tissue samples. Most importantly, this 5-gene PDAC classifier discriminates between PDAC and pancreatitis and between PDAC precursor lesions and healthy pancreas. The 5-gene PDAC classifier encodes proteins involved in PDAC pathogenesis, such as TMPRSS4 and ECT2, that are overexpressed even at the earliest stages of PDAC development, suggesting causality during early PDAC development.

The 5-gene PDAC classifier performed well across multiple microarray platforms from different laboratories and cross-species, using either whole tissue or microdissected tissue. While over 2500 candidate biomarkers have been associated with PDAC and some of these candidates are in various stages of evaluation, only CA19-9 is FDA-approved for PDAC [28]. Nevertheless, CA19-9 does not provide an accuracy high enough for screening, particularly for early detection or risk assessment. Currently, no diagnostic or predictive gene or protein expression biomarkers that accurately discriminate between healthy patients, benign, premalignant and malignant disease have been extensively validated. The goal of this study was to identify a biomarker panel with greater sensitivity and specificity compared to the primary biomarker currently used for PDAC, CA19-9. Our data suggest that a multiplex panel of biomarkers, rather than a single biomarker, is more likely to improve the specificity and selectivity for accurate detection of PDAC. Compared to the sensitivity of 79-81% and specificity of 82-90% for CA19-9 the sensitivity of our 5-gene PDAC classifier is superior [>85%] and with equal or better specificity [29].

We converted the in silico validation of publicly available transcriptome datasets into a qRT-PCR based assay for FFPE samples and confirmed in 22 pairs of retrospective patient samples that the 5-gene panel clearly discriminates between PDAC and pancreatitis or healthy pancreas. Importantly, expression of three of the five genes, POSTN, SERPINB5 and TMPRSS4, apparently increases gradually from healthy pancreas to pancreatitis and further rises between pancreatitis and PDAC. These three genes, thus, may reflect different pathophysiological functions from the two genes, AHNAK2 and ECT2 that are increased to a similar degree in PDAC when compared to pancreatitis or healthy pancreas. Differential diagnosis between PDAC and pancreatitis is critical, since patients with chronic pancreatitis are at increased risk of PDAC development and pathological discrimination between PDAC and pancreatitis can be challenging for definitive diagnosis of PDAC. Recent advances in endoscopic ultrasound (EUS) have yielded improved sensitivity for PDAC identification. Nevertheless, differentiating between PDAC and benign disease remains operator-dependent and sometimes challenging, frequently requiring multiple biopsies and procedures. Even after two EUS fine needle aspiration (FNA) biopsy procedures, diagnosis remains ambiguous for a subset of patients with a pancreatic abnormality [30], particularly in the setting of pancreatitis. This may result in diagnostic uncertainty and delays in potentially curative treatment. Thus, a key unmet medical need with immediate clinical utility is an effective cell-based diagnostic test that accurately differentiates between PDAC and non-malignant pancreatic disorders such as chronic pancreatitis.

Pre-operative diagnosis of pancreatic cancer is often limited by the performance of Endoscopic ultrasound-guided fine-needle aspiration (EUS-FNA), a common diagnostic approach that may be inconclusive in up to 20% of cases and for which inter-observer reliability is suboptimal even for biopsy evaluation [31–36]. More specifically, a wide range of performance parameters have been reported for malignancy diagnosis on cytopathologic analysis of EUS FNA: sensitivity from 73% to 94%, accuracy from 78% to 95% and a negative predictive value from 40% to 85% when specificity approaches 100% [31–36]. Therefore, improved performance for the diagnosis of pancreatic lesions on aspiration or biopsy tissue is critical to early and effective treatment. The 5-gene PDAC classifier described in this manuscript may be first applied as a FNA-based real time PCR test to complement the pathologist and enhance positive and negative predictive value in diagnosing pancreatic cancer and differential diagnosis.

Moreover, due to widespread use of abdominal cross-sectional imaging, pancreatic cysts are detected in ~2% of all patients who undergo abdominal imaging with computed tomography and ~15% of all patients who undergo an abdominal MRI. Once imaged, pancreatic cysts are frequently biopsied and classified as PanINs, MCNs and IPMNs. However, the guidelines with regards to monitoring and treatment of these cystic lesions remain unclear and no definitive diagnosis about malignancy or risk of malignancy can be made.

Frequently, EUS combined with FNA is used to visualize the pancreas from the duodenum or stomach but is sometimes challenged with diagnostic accuracy in detecting malignant lesions under 3 cm even by skilled clinicians. However, EUS-FNA has less than optimal accuracy for differentiating pancreatic cancer from chronic pancreatitis. Since only a small minority of these cysts, but still significant number, subsequently progress to cancer, there may be needless over- or under-treatment, and morbidity. Our 5-gene classifier may be able to be applied in the setting where FNAs are taken from cysts or other suspicious pancreatic lesions for differential diagnosis.

Above all, the 5-gene PDAC classifier accurately distinguishes premalignant and malignant pancreatic lesions such as PanIN, IPMN with low- to intermediate grade dysplasia, IPMN with high-grade dysplasia and IPMN with associated invasive carcinoma from healthy pancreas. In this context it is reassuring to observe that the 5-gene PDAC classifier performs equally well in the PDX1-Cre;LSL-Kras^G12D^ mouse model of PDAC that phenocopies the human disease and allows evaluation of the different developmental stages of PDAC development, including PanIN, that are typically not detected and sampled in humans [37]. We discovered that all 5 genes are overexpressed already in PanIN, indicating that these 5 genes become dysregulated very early during PDAC development and could indeed assist in early detection of PDAC. IPMNs and other pancreatic cysts are frequent events in older patients (in some autopsy studies up to 24%). Imaging improvements have resulted in an increasing number of asymptomatic, incidentally discovered pancreatic cysts [38]. While many are benign, MCNs and IPMNs have a significant incidence (2.4-13.5%) of either harboring malignant cells or progressing towards invasive cancer [39]. Consequently, pancreatic cysts cannot be ignored; however, current diagnostic tools are limited for accurately predicting their malignant potential. Survival upon surgical resection of non-malignant lesions is close to 100% and after malignant transformation drops to 55-60% [40]. Resection of all pancreatic cysts is difficult due to relatively high rates of mortality (1-6%) and morbidity (35-51%) in elderly patients. A key challenge is how to prioritize the cysts that can be ignored or followed versus the ones that should be surgically resected. An early detection marker, one able to detect PDAC precursor lesions (IPMN, PanIN) with early malignant transformation or high risk for malignant transformation, would increase the likelihood of identifying such patients that may have localized disease amendable to curative surgery. Better diagnosis of borderline and invasive IPMNs and MCNs would be highly significant, and enable patients to choose the most appropriate course of action; this 5-gene PDAC classifier may provide such a risk assessment. Discovery and validation of a distinct set of sensitive and specific biomarkers for risk-stratifying patients at high risk for developing PDAC would eventually enable routine screening of high risk groups (i.e., incidental detection of pancreatic lesions, family history of PDAC, hereditary syndromes (VHL), chronic pancreatitis, type 3c diabetes, smokers, BRCA2 carriers, etc).

While other studies have performed meta-analysis of transcriptome data for PDAC, such as a recent meta-analysis study that identified more than 800 genes that are overexpressed in PDAC [41], none of these studies including this latest report attempted to select any genes as PDAC predictors. In contrast, there has been significant progress in identifying circulating miRNAs that distinguish PDAC from chronic pancreatitis and healthy patients in plasma and bile [42]. A 5 miRNA panel diagnosed PDAC with 95% sensitivity and specificity in a cohort that included healthy, chronic pancreatitis and PDAC patients [42]. However, there is no evidence whether these miRNAs would diagnose early stages of PDAC.

The set of PDAC biomarkers identified in this study may be regulated by a common set of upstream regulatory transcription factors. However, based on available data and Ingenuity upstream regulator analysis no common upstream regulator or transcription factor was identified by us. This is no surprise, since AHNAK2 and TMPRSS4 have rather limited information available. Using the Transfac database we have analyzed the promoter regions between −2000 and +100 for the 5 genes for common transcription factor binding sites. Transfac analysis identified 6 transcription factors (ZNF333, Ikaros, ING4, MZF-1, CRX, NF-AT1) with predicted binding sites in 4 out of the 5 genes, suggesting that these transcription factors may be common to these PDAC classifier genes (data not shown). Nevertheless, further studies are needed to explore the potential role of these transcription factors in controlling expression of these genes in PDAC.

To determine whether the set of biomarkers encoded by our PDAC classifier may also reflect key pathophysiological pathways associated with PDAC development or progression that may be candidate therapeutic targets (i.e., similar to value of Her2/neu as a biomarker and therapeutic target), we reviewed available public data for the classifier genes.

The 5-gene classifier includes two genes, TMPRSS4 and POSTN, previously identified as candidate PDAC biomarkers further validating our innovative meta-analysis strategy to select significant PDAC biomarkers. Several genes of our 5-gene classifier have been linked to tumorigenesis, indicating a causal role in PDAC development and progression.

Most of the classifier genes (TMPRSS4, POSTN, SERPINB5) have been linked to migration, invasion, adhesion and metastasis of PDAC or other cancers, specifically associated with extracellular matrix and tumor microenvironment. This may not be surprising due to the dense, desmoplastic stroma associated with PDAC that may prevent efficient drug delivery [43]. However, these biological functions would be anticipated to be involved in PDAC progression rather than early stages of PDAC development. To explore this aspect in more detail we evaluated the expression levels of these “PDAC progression” genes in the transcriptome datasets comparing PDAC precursors (LIGD-IPMN, HGD-IPMN) and InvCa-IPMN to normal pancreas, and PDAC vs. PanIN vs. healthy pancreas in the GEM model) (Figure 4) [15]. TMPRSS4, SERPINB5, ECT2, and AHNAK2 are overexpressed in LIGD-IPMN, HGD-IPMN, and InvCa-IPMN as well as in PanINs, as compared to normal pancreas, demonstrating that enhanced expression of multiple genes linked to metastasis and PDAC progression occurs early on during malignant development. This analysis indicates that the PDAC classifier may reflect some driving early defects during PDAC development.

No details about AHNAK2 function are available, but its closest relative AHNAK is involved in cancer migration and EMT, providing support that AHNAK2 may elicit similar features [44]. Periostin (POSTN), frequently overexpressed in melanoma, pancreatic, esophageal, prostate, and liver cancer [45–47], promotes invasiveness and metastasis of PDAC [48] and other cancer types [49]. Maspin (SERPINB5) overexpression correlates with increased metastasis and poor outcome in PDAC and gastric cancer [50–52]. But SERPINB5 is regarded as a metastasis suppressor in breast and colorectal cancer [50]. For PDAC, conflicting roles of SERPINB5 have been described: SERPINB5-transfected PDAC cells exhibit reduced invasive ability [53], but SERPINB5 overexpression in PDAC is an independent adverse prognosticator for postoperative survival [51]. The detection of SERPINB5 in high-grade PanIN and PDAC and its lack of expression in normal pancreatic tissues and chronic pancreatitis suggest that SERPINB5 upregulation occurs early during the multi-step progression of PDAC [53, 54]. The oncogene ECT2 is overexpressed in several cancer types including PDAC, and correlates with poor outcome in glioma and gastric cancer [26]. We now provide the first evidence that ECT2 may play a role in PDAC viability and soft agar growth.

TMPRSS4, a protease of the Type II transmembrane serine protease (TTSP) family, is highly expressed in pancreatic, thyroid, lung, gastric, cervical, breast, and colorectal cancer tissues and directly correlates with poor outcome [19–25]. TMPRSS4 expression correlates with the metastatic potential of several cancer cell lines, and our cell-based studies demonstrate that TMPRSS4 induces migration, invasion and anchorage-independent growth [20, 24, 25]. Moreover, we provide the first qRT-PCR-based analysis for enhanced TMPRSS4 expression in microdissected PDAC tissue as compared to pancreatitis or healthy pancreas. TMPRSS4 promotes invasion, migration and metastasis of colorectal and lung cancer cells by facilitating an EMT [24, 25]. Multiple downstream signaling pathways, including focal adhesion kinase (FAK), extracellular signal-regulated kinase (ERK), Akt, Src and Rac1, and integrin alpha5 expression are activated by TMPRSS4 expression in lung and colon cancer cells [20, 25, 55].

In conclusion, we have identified a new multiplex panel of biomarkers for early detection of PDAC that can be developed as a diagnostic assay, facilitating early diagnosis and screening for malignant transition of potential PDAC precursor lesions and for high risk patient groups. Since the number of samples evaluated in this study for discrimination of early stages (PanIN, IPMN with dysplasia) of PDAC is relatively small, we will have to further establish the accuracy on larger numbers of such specimens. Our data combined with the supportive published evidence suggest that the five biomarkers identified in our PDAC classifier can be exploited for development of new drugs targeting these markers, since these genes are overexpressed in precursor lesions and have a strong rationale for involvement in PDAC tumorigenesis and metastasis.

## MATERIALS AND METHODS

### Meta-analysis to identify optimal PDAC biomarker panel

#### Dataset identification

Four training sets [GSE18670, E-MEXP-950, GSE15471, GSE16515] and nine independent validation sets [GSE32676, GSE28735, GSE11838, GSE9599, E-MEXP-2894, E-TABM-145, E-MEXP-1121, GSE19650, GSE12630] of transcriptome data for human pancreas specimens and one dataset of transcriptome data from the PDX1-Cre; LSL-Kras^G12D^ genetically engineered mouse (GEM) model of PDAC (GSE33322) were selected from publicly available microarray repositories as described in Supplementary Material and Methods.

#### Quality control and outlier analysis

Stringent quality control and outlier analysis was performed on all datasets used for training and validation to remove low quality arrays from the meta-analysis as described in Supplementary Material and Methods.

#### Mapping of platform specific identifiers to entrez gene IDs

To facilitate collation of the differentially expressed genes identified by analysis of individual datasets, the probe-level identifiers associated with each dataset were annotated with corresponding gene-level identifiers as described in detail in Supplementary Material and Methods.

#### Pre-processing and normalization of microarray datasets

Potential bias introduced by the range of methodologies used in the original microarray studies, including various experimental platforms and analytic methods, was controlled by applying a uniform normalization, preprocessing and statistical analysis strategy to each dataset using the Frozen Robust Multi-array Average (fRMA) algorithm with rma background correction and Z-score as described in Supplementary Material and Methods.

#### Differential gene expression analysis

For training set differential expression analysis, the two sample classes were normal pancreas (NP) and PDAC and the null hypothesis was “no difference in gene expression exists between the NP and PDAC sample classes”. The differentially expressed transcripts were identified using the linear model microarray analysis software package (LIMMA) and on the basis of absolute fold change of at least 1.5 and Benjamini and Hochberg corrected p-value <.05. Differentially expressed genes with concordant directionality (upregulation or downregulation) in three out of four datasets were used for training the PDAC classifier.

#### Training and independent validation of PDAC classifier using support vector machine

The 409 genes differentially expressed in at least 3 out of 4 datasets were used for classifier generation by implementing the Support Vector Machines (SVM) approach using Bioconductor and using 0 as the threshold. Classifiers were trained using normalized, preprocessed gene expression values. Performance of classifiers in the training sets was evaluated using internal Leave-One-Out Cross-Validation (LOOCV). The performance of classifiers was measured using threshold-dependent (e.g. sensitivity, specificity, accuracy) and threshold-independent receiver operating characteristic (ROC) analysis. In ROC analysis, the area under the curve (AUC) provides a single measure of overall prediction accuracy. We developed biomarker panels ranging from 2 to 40 genes to develop highly accurate biomarker panels with a minimum number of genes. The biomarker panel with the highest performance in the training sets was chosen for assessment of predictive power in 9 independent validation datasets using threshold-dependent and -independent (AUC) measures. For independent validation the same threshold selected for the training set was applied.

To further compare the performance of actual classifiers with random classifiers we performed randomization analysis. 1000 random classifiers of the size of actual classifiers were developed, and their performances were compared. Statistical testing (p-value) evaluated the hypothesis that random classifiers had the same or better performance than the actual classifier.

#### Correlative laboratory evaluation

Antibodies, reagents, lentiviral production and infection, cell culture, cell-based assays (proliferation, migration, invasion, soft agar growth), and Western blot analysis are described in Supplementary Material and Methods.

#### Quantitative real-time PCR (qRT-PCR) analysis of FFPE samples

qRT-PCR analysis was performed on RNA isolated from core punches, restricted to tumor regions that a fellowship-trained, gastrointestinal and hepato-pancreato-biliary pathologist (EUY) marked as PDAC, pancreatitis or healthy pancreas, from human formalin-fixed paraffin embedded (FFPE) tissue obtained from 22 PDAC patients (9 well-differentiated, 9 moderately-differentiated, 3 poorly differentiated, 1 other) as described in detail in Supplementary Material and Methods.

## SUPPLEMENTARY MATERIAL AND METHODS, REFERENCES, TABLE AND FIGURES


