# Supplementary material for: Meta-analysis of transcriptome data identifies a novel 5-gene pancreatic adenocarcinoma classifier
Source: Oncotarget. 2016 Mar 16;7(17):23263–81. doi: 10.18632/oncotarget.8139 (PMC5029625; doi:10.18632/oncotarget.8139)
Supplement: Supplementary file 1 [file oncotarget-07-23263-s001.pdf]

Meta-analysis of transcriptome data identifies a novel 5-gene pancreatic adenocarcinoma classifier

Supplementary Material and Methods

Meta-analysis to identify optimal PDAC biomarker panel

Dataset identification

The literature and the publicly available microarray repositories (ArrayExpress, Gene Expression Omnibus (GEO), ONCOMINE, and Stanford Microarray Database [SMD]) were searched for gene expression studies of human pancreatic specimens. The selected datasets were divided into four training sets and nine independent validation sets. Each training dataset (GSE18670, E-MEXP-950, GSE15471, GSE16515) included a minimum of four samples of normal pancreas (NP) and a minimum of four samples of PDAC. GSE18670 and E-MEXP-950 was derived from microdissected tissue, whereas GSE15471 and GSE16515 used RNA isolated from whole tissue pieces. The nine validation sets included three datasets of PDAC and normal pancreas (GSE32676, GSE28735, GSE11838), two datasets of PDAC only (GSE9599, E-MEXP-2894), one dataset of normal pancreas only (E-TABM-145), one dataset of pancreatitis and PDAC (E-MEXP-1121), one dataset of PDAC and several other cancer types (GSE12630), and one dataset of normal pancreas and PDAC precursors (GSE19650).

In addition, one gene expression dataset of PDAC, PanIN and healthy pancreas derived from the PDX1-Cre; LSL-Kras^{G12D} GEM mouse model of PDAC (GSE33322) was available in the public databases.

Quality Control and Outlier analysis

Stringent quality control and outlier analysis was performed on all datasets used for training and validation to remove low quality arrays from the meta-analysis. The technical quality of arrays was determined on the basis of background values, percent present calls, scaling factors, and 3'-5' ratio of β-actin and GAPDH using various bioconductor packages (1, 2). The arrays with high quality were subjected to outlier analysis using array intensity distribution, principal component analysis, array-to-

array correlation and unsupervised clustering. The samples that were identified to be of low quality or outliers were eliminated from the meta-analysis.

Mapping of platform specific identifiers to Entrez Gene IDs

To facilitate the collation of the differentially expressed genes identified by analysis of individual datasets, the probe-level identifiers associated with each dataset were annotated with corresponding gene-level identifiers. GeneIDs were used in all subsequent analyses to map genes across the datasets to avoid ambiguity from non-unique gene identifiers. For Affymetrix GeneChip data, Affymetrix probe set IDs were annotated using the appropriate microarray chip annotation package (http://www.bioconductor.org/packages/release/AffymetrixChip.html) and the annotate package (http://www.bioconductor.org/packages/2.4/bioc/html/annotate.html) or biomaRt package available through Bioconductor. Because each human Entrez Gene ID (GeneID) is unique for a **single** gene and each gene can only map to one GeneID, the four lists of differentially expressed genes from the four training sets were combined using the GeneIDs that correspond to the probe IDs specific to a given microarray platform. Affymetrix probe set IDs that could not be mapped to an Entrez Gene ID (GeneID) were removed from the gene lists.

Pre-processing and normalization of microarray datasets:

Potential bias introduced by the range of methodologies used in the original microarray studies, including various experimental platforms and analytic methods, was controlled by applying a uniform normalization, preprocessing and statistical analysis strategy to each dataset.

Each Affymetrix dataset was normalized from raw data (.cel files), when available, using the Frozen Robust Multi-array Average (fRMA) algorithm with rma background correction implemented through functions provided by the Bioconductor package affy (3). fRMA is a microarray-preprocessing algorithm

that utilizes information from large publicly available microarray databases to pre-compute and freeze estimates of probe-specific effects and variances. The frozen fRMA data is updated with information from new array datasets to provide a normalized summary of the combined data. When the probe-level data contained in .cel files was not available, we used the gene expression data matrix (GEDM) of Affymetrix average difference intensities. The normalized datasets were further standardized using Z-score to reduce the batch effects among different datasets (4).

Differential gene expression analysis

For training set differential expression analysis, the two sample classes were normal pancreas (NP) and PDAC and the null hypothesis was "no difference in gene expression exists between the NP and PDAC sample classes". To identify differentially expressed genes, a linear model was implemented using the linear model microarray analysis software package (LIMMA) (5). The differentially expressed transcripts were identified using LIMMA, which estimates the differences between Normal and Cancer samples by fitting a linear model and using an empirical Bayes method to moderate standard errors of the estimated log-fold changes for expression values from each probe set. In LIMMA, all probes were ranked by *t* statistic using a pooled variance, a technique particularly suited to small numbers of samples per phenotype. The differentially expressed probes were identified on the basis of absolute fold change and Benjamini and Hochberg corrected P value (6). The genes with multiple test corrected P value <.05 and fold change (FC) of at least 1.5 were considered as differentially expressed. The genes that were found to be differentially expressed and with concordant directionality (upregulation or downregulation) in three out of four datasets were used for training the PDAC classifier.

Hierarchical clustering analysis of mouse GEM PDAC model microarray dataset

To evaluate the cross-species differential expression of the 5-gene PDAC classifier in a GEM mouse model of PDAC we performed unsupervised analysis using hierarchical clustering analysis (HCA) on the

GSE33322 dataset. The HCA analysis was performed using Pearson correlation matrices with complete-linkage method.

Correlative Laboratory Evaluation

Antibodies and reagents

Dulbecco's Modified Eagle's Medium (DMEM), phosphate-buffered saline (PBS), fetal bovine serum (FBS), trypsin ethylenediamine tetraacetic acid (EDTA), glutamine, penicillin streptomycin, and culture supplements were purchased from Gibco-BRL Life Technologies (Palto Alto, CA, US). TMPRSS4 antibodies used in Western blots were purchased from Sigma (MO, US). All other reagents and materials were purchased from Thermo Fisher Scientific (GA, US).

Cell culture

Capan-1, BxPC-3, MIAPaCa-2, Panc-1, ASPC1, PL45 and HPDE cells were purchased from American Type Culture Collection (Rockville, MD, US). These cells were maintained in Dulbecco's modification of Eagle's medium (DMEM) containing 10% fetal bovine serum, 1% penicillin/streptomycin, and 1% glutamine. Cell lines were cultured in BD Primaria tissue culture dishes, with dimensions of 100x20 mm at 37°C with 5 % CO_2 in a humidifier incubator and carried at 2.0×10^6 cells/ml, passaging two to three times weekly as needed. Cells were pelleted by centrifugation at 2,500 rpm for 8 min at 4°C and resuspended in fresh complete media in tissue culture plates 24 hrs before use in experiments to avoid any confounding gene expression that might occur because of handling. Confluent cells were harvested by trypsinization with 0.05 % trypsin and 0.02 % EDTA, pelleted by centrifugation at 2,500 rpm for 8 min at 4°C, and resuspended in fresh complete DMEM media and plated in BD Primaria tissue culture dishes 24 hrs before use in experiments.

Lentiviral production and infection

Lentiviral shRNAs targeting TMPRSS4 (shTMPRSS4) and ECT2 (shECT2) were obtained from Harvard Medical School (Boston, MA). The lentivirus was packaged by co-transfection of 293T cells with the shRNA expression vector, VSV-G (vesicular stomatitis virus-glycoprotein), and delta-VPR plasmids at the ratio of 1:0.9:0.1, using lipofectamine 2000 (Invitrogen, USA). Forty-eight hours after transfection, the supernatants containing lentiviral particles were harvested and titering was done using Hela cells.

Capan-1 and BxPC-3 cells were plated in 10 cm dishes until 80% confluence. The day of infection, media was removed and replaced with 8 ml of complete media supplemented with polybrene (8 ug/ml) into each plate. 250 μl of lentivirus (shTMPRSS4, shECT2, shGFP or a scrambled shRNA control) was added to each plate and incubated for 24 hours. Cells were left to recover from infection for 24 hours before initiating selection with puromycin 3ug/ml for three days.

Proliferation Assay

Cell viability was indirectly assessed with a colorimetric, (3-(4, 5-dimethylthiazol-2-yl)-5-(3-carboxymethoxyphenyl)-2-(4-sulfophenyl)-2H-tetrazolium) (MTS) assay obtained from Promega. In brief, 5 X 10^3 cells/well (100 μl/well) were plated on Fisher brand 96 well cluster dishes and infected with shTMPRSS4 or shECT2. After 24 hours of incubation, DMEM medium was removed and followed by the addition of 20 μl of MTS solution to each well. The 96 well plates were placed in an incubator at 37°C in 5% CO_2.

The absorbance of the solution was measured in a spectrophotometer (Bio-Rad Model 550, Bio-Rad Laboratories, Inc., Hercules, CA, USA) using a test wavelength of 540 nm.

Migration and invasion assays

Cell migration and invasion with shTMPRSS4 or shECT2 were tested using a modified Transwell chamber migration assay (8-μm pore size membrane, BD Biosciences) or invasion assay (Matrigel-coated membrane, BD Biosciences). Cells (25 × 10^4) were seeded in serum-free medium into the upper

chamber and allowed to migrate or invade toward 10% FCS as a chemoattractant in the lower chamber for 16 h. Cells in the upper chamber were carefully removed using cotton buds, and cells at the bottom of the membrane were fixed and stained with crystal violet. Quantification was done by counting the stained cells.

Soft agar colony formation assay

The soft agar assay was used to determine colony formation of the cells for detection of malignant cell transformation. Briefly, $1x10^5$ shTMPRSS4 or shECT2 infected cells were added to the top layer soft agar mix in six well plates. The six well plates were stored in the incubator between 14-20 days. A few drops of DMEM were added every two to three days to keep the plate moist. After incubation colony formation was determined using a P-Iodonitrotetrazolium staining dye.

Western blot analysis

For Western blot analysis, $1.0x10^6$/ml of cells infected with shTMPRSS4 were cultured. Cells were harvested and pelleted in an Eppendorf microcentrifuge (1200 x *g*, 5 min, 4°C), washed in 1xPBS and resuspended in a cell lysis buffer containing 20 mM Tris (pH 8.0), 0.5% (w/v) Nonidet P-40, 1 mM ethylenediamine tetraacetic acid (EDTA), 1 μg/ml leupeptin, 1μg/ml pepstatin, 1 mM dithiothreitol, 1 mM PMSF and 0.1 M NaCl. After 20 min incubation at 4°C, supernatants were clarified by centrifugation (8000 x *g*, 5 min, 4°C) and their total protein concentration was determined by the method of Bradford and Lowry using Bio-Rad Protein Assay reagents in a microtiter assay. Total cellular protein (40μg) was electrophoresed on a sodium dodecyl sulphate polyacrylamide gel (SDS-PAGE) and then transferred to a polyvinylidine difluoride membrane (Amersham, IL, USA) by electroblotting overnight in 25 mM Tris (pH 8.3), 192 mM glycine, 20% (v/v) methanol, at 15 V, 100 mA, 4°C. The membranes were blocked with 10% (w/v) electrophoresis-grade biotin-depleted non-fat dry milk (Bio-Rad) in 1xPBS, rinsed in PBS, probed with monoclonal mouse anti-human TMPRSS4, at a 1:250 dilution and washed 3X in PBS. The secondary antibody was HRP-conjugated goat anti-mouse or anti-rabbit whole IgG used at 1:5000

dilution (Transduction Laboratories, CA, USA) for one hour at room temperature. The protein bands were then visualized using an enhanced chemiluminescence (ECL) detection system (Amersham Bioscience, NJ, USA).

Quantitative real-time PCR (qRT-PCR) analysis of FFPE samples

With institutional review board (IRB) approval, human formalin-fixed paraffin embedded (FFPE) tissue was obtained from 22 PDAC patients who underwent primary surgical resection (pancreaticoduodenectomy or partial pancreatectomy). The original slides (5 μm thickness) of FFPE tissue that were prepared and stained with hematoxylin and eosin were reviewed by a fellowship-trained, gastrointestinal and hepato-pancreato-biliary pathologist (EUY). 9 well-differentiated, 9 moderately-differentiated, 3 poorly differentiated and 1 other (mucinous adenocarcinoma/colloid carcinoma) PDAC samples and their respective background, non-neoplastic pancreatic parenchyma (9 with no significant pathologic abnormality and 13 with pancreatitis) were selected. Regions of neoplastic and background pancreatic parenchyma were designated for analysis (areas at least 64 mm^2 in size were outlined with permanent marker).

After matching the tissue block with the H&E stained slide, core punches, restricted to tumor regions that the pathologist marked as PDAC, pancreatitis or healthy pancreas, were extracted from the FFPE block. A 2.5 mm biopsy punch was used to punch three cores from each sample for RNA extraction. Total RNA was isolated using the RecoverAll™ Total Nucleic Acid Isolation Kit (Ambion) after pooling the three cores for each sample.

1 ug of total RNA extracted from the FFPE tissue samples was reverse transcribed using the High-Capacity cDNA Reverse Transcription Kit (Life Technologies, Grand Island, NY) according to the manufacturer's instructions. TaqMan RT-PCR reactions were performed in duplicates in 96-well reaction plates in a total volume of 20 µL using TaqMan Fast Advanced Master Mix (Life Technologies). The following TaqMan Gene Expression Assays (Life Technologies) were mixed with template cDNA: GAPDH, POSTN, SERPINB5 AHNAK2, TMPRSS4, ECT2. qRT-PCR Reaction plates were run

on the Applied Biosystems StepOne Plus Real-Time PCR System with the following profile: 50°C hold for 2 minutes, 95°C for 20 seconds followed by 45 cycles of 95°C for 1 second and 60°C for 20 seconds. The analysis was done using StepOne™ Software v2.2.2. For normalizing target gene expression, GAPDH mRNA (house keeping gene (HKG)) expression was used. The expression measurements were performed on the RNA in duplicate. Relative quantity values were calculated for each tumor sample using the matched pancreatitis or normal tissue as the baseline,

Taqman primers for POSTN (Assay ID: Hs01566734_m1), SERPINB5 (Assay ID: Hs00985285_m1), AHNAK2 (Assay ID: Hs00292832_m1), TMPRSS4 (Assay ID: Hs00212669_m1) and ECT2 (Assay ID: Hs00978168_m1) were obtained from Life Technologies.

References

1. R_Development_Core_Team. R: A language and environment for statistical computing.2009.

2. Wilson CL, Miller CJ. Simpleaffy: a BioConductor package for Affymetrix Quality Control and data analysis. Bioinformatics. 2005;21:3683-5.

3. McCall MN, Bolstad BM, Irizarry RA. Frozen robust multiarray analysis (fRMA). Biostatistics (Oxford, England). 2010;11:242-53.

4. Le Cao KA, Rohart F, McHugh L, Korn O, Wells CA. YuGene: a simple approach to scale gene expression data derived from different platforms for integrated analyses. Genomics. 2014;103:239-51.

5. Smyth GK. Linear models and empirical bayes methods for assessing differential expression in microarray experiments. Statistical applications in genetics and molecular biology. 2004;3:Article3.

6. Benjamini Y, Hochberg Y. Controlling the false discovery rate: a practical and powerful approach to multiple testing. Journal of the Royal Statistical Society: Series B. 1995;57:11.

Supplementary Table S1. Significantly differentially Expressed genes identified in different datasets

Dataset	Upregulated Genes	Downregulated Genes
Set 1	449	250
Set 2	36	54
Set 3	4105	6064
Set 4	2418	3751

A)

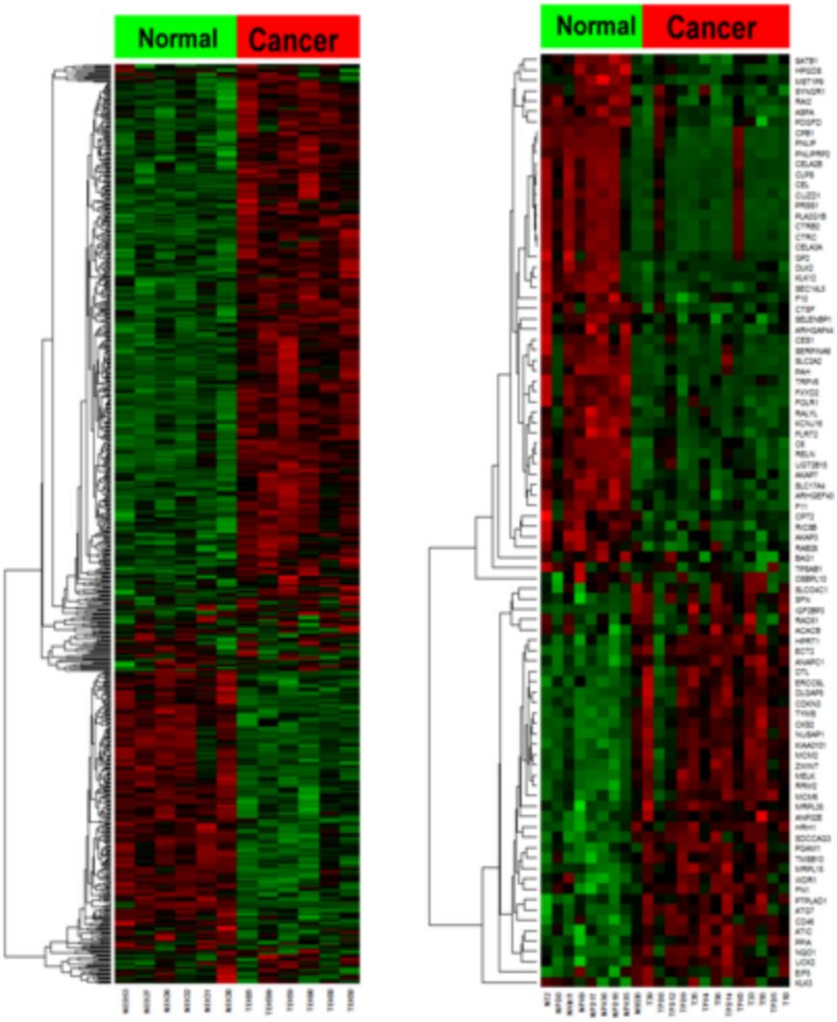
Normal
Cancer
Normal
Cancer

B)

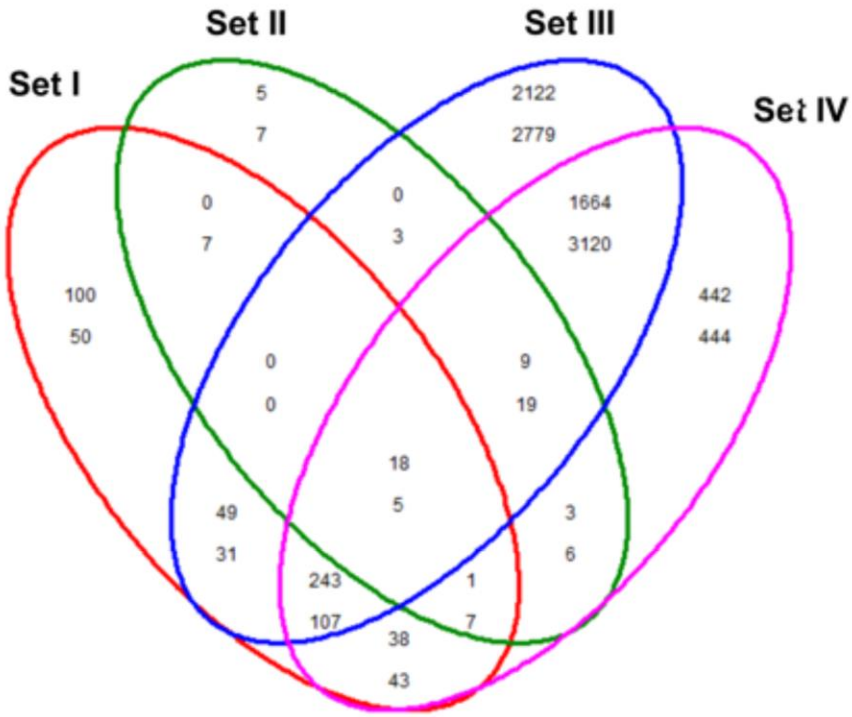
Set I
Set II
Set III
Set IV
5
7
2122
2779
0
7
0
3
1664
3120
100
50
442
444
0
0
9
19
18
5
49
31
3
6
243
107
1
7
38
43

C)

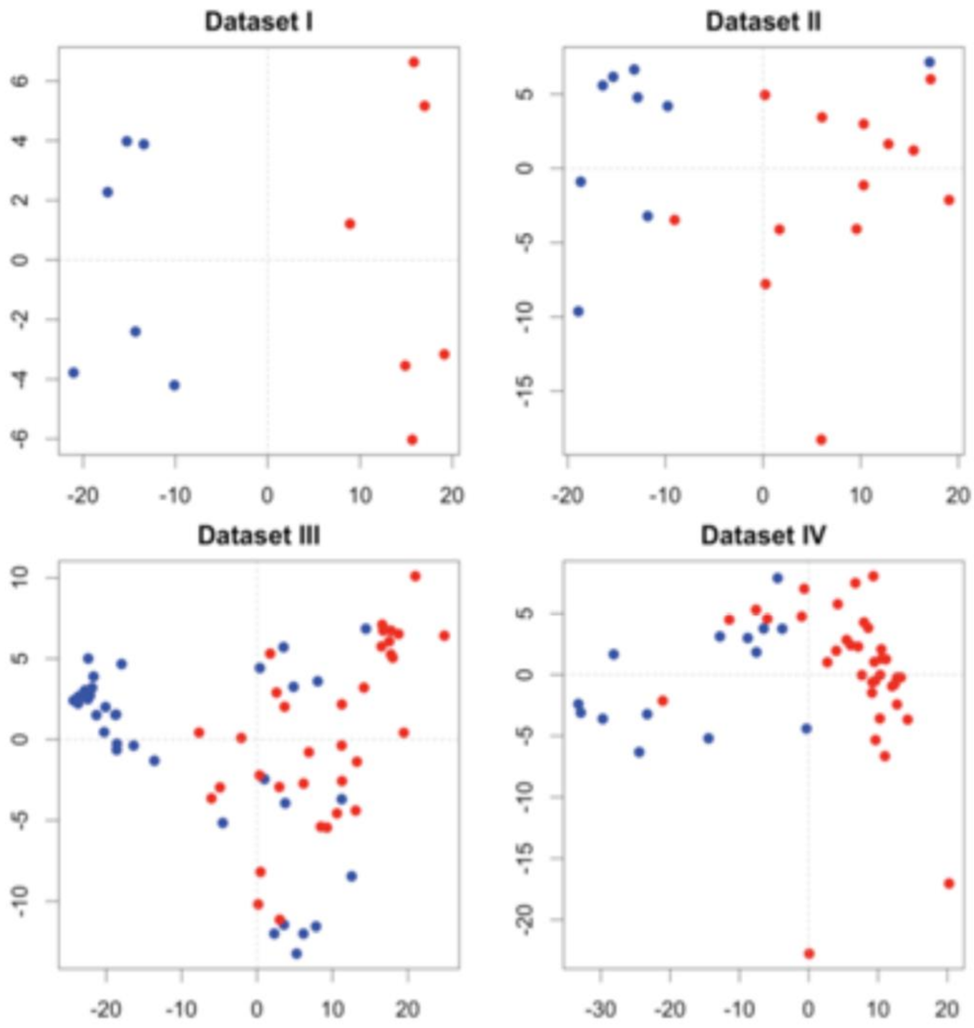

Supplementary Figure S1. Identification of significantly differentially expressed genes using empirical Bayes approach from uniformly normalized and transformed data. A) Heatmaps for genes differentially expressed in PDAC for two of the four training datasets. Red = upregulated, Green = downregulated. B) Venn diagram of the four training datasets for the differentially expressed genes. 409 genes with concordant directionality are common to at least 3 of the 4 datasets. C) PCA plots for each dataset using the 409 meta-signature genes. Differentially expressed genes are defined by an associated limma p-value less than 0.05 with Benjamini and Hochberg method for multiple comparison correction to control FDR.

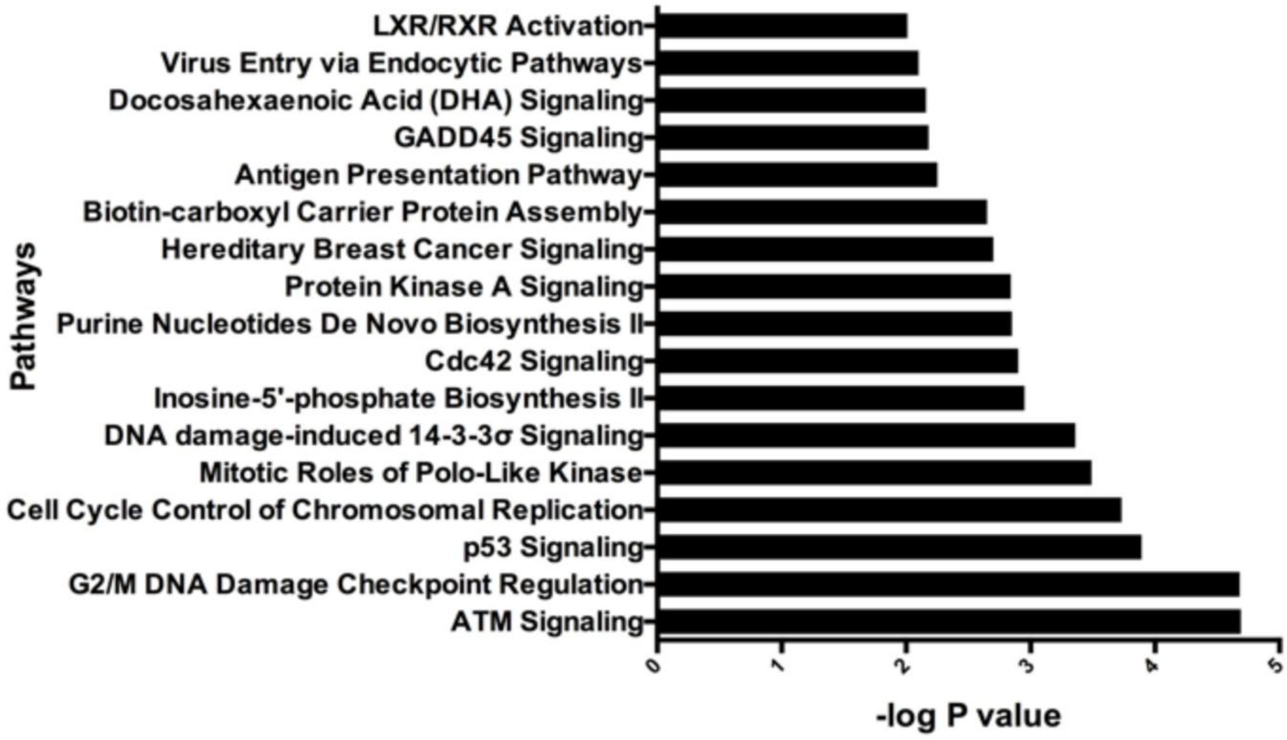

Supplementary Figure S2. Canonical pathway analysis of the 409 PDAC-specific genes using IPA.

POSTN

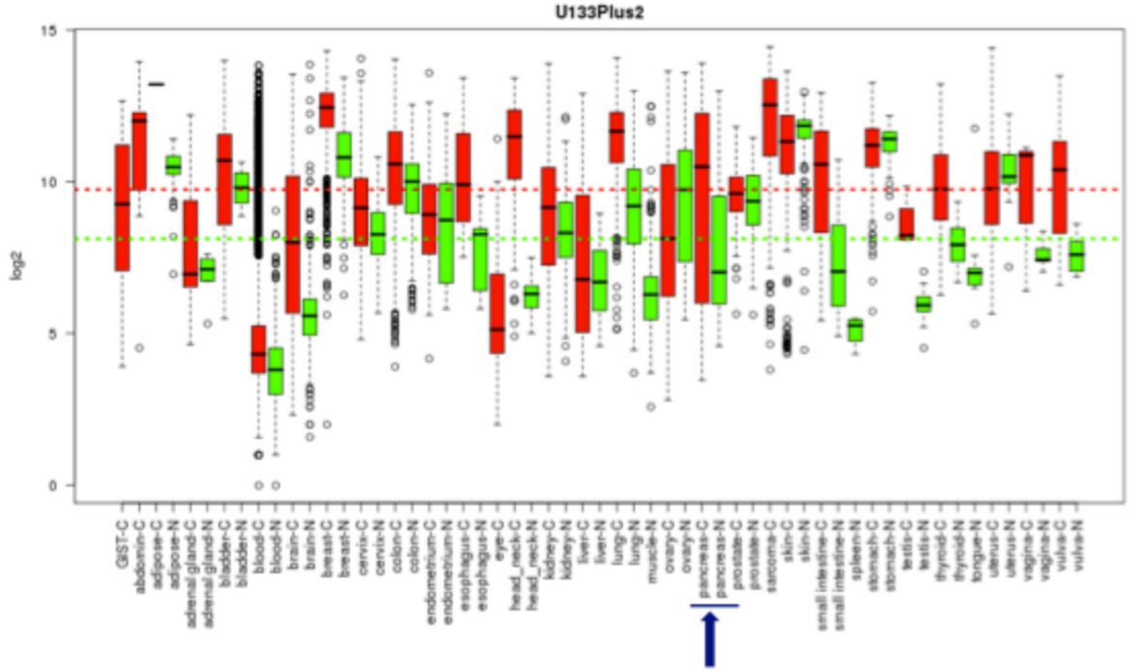
U133Plus2
log2
0
5
10
15
GIST-C
abdomin-C
adipose-C
adipose-N
adrenal gland-C
adrenal gland-N
bladder-C
bladder-N
blood-C
blood-N
brain-C
brain-N
breast-C
breast-N
cervix-C
cervix-N
colon-C
colon-N
endometrium-C
endometrium-N
esophagus-C
esophagus-N
eye-C
head_neck-C
head_neck-N
kidney-C
kidney-N
liver-C
liver-N
lung-C
lung-N
muscle-N
ovary-C
ovary-N
pancreas-C
pancreas-N
prostate-C
prostate-N
sarcoma-C
skin-C
skin-N
small intestine-C
small intestine-N
spleen-N
stomach-C
stomach-N
testis-C
testis-N
thyroid-C
thyroid-N
tongue-N
uterus-C
uterus-N
vagina-C
vagina-N
vulva-C
vulva-N

ECT2

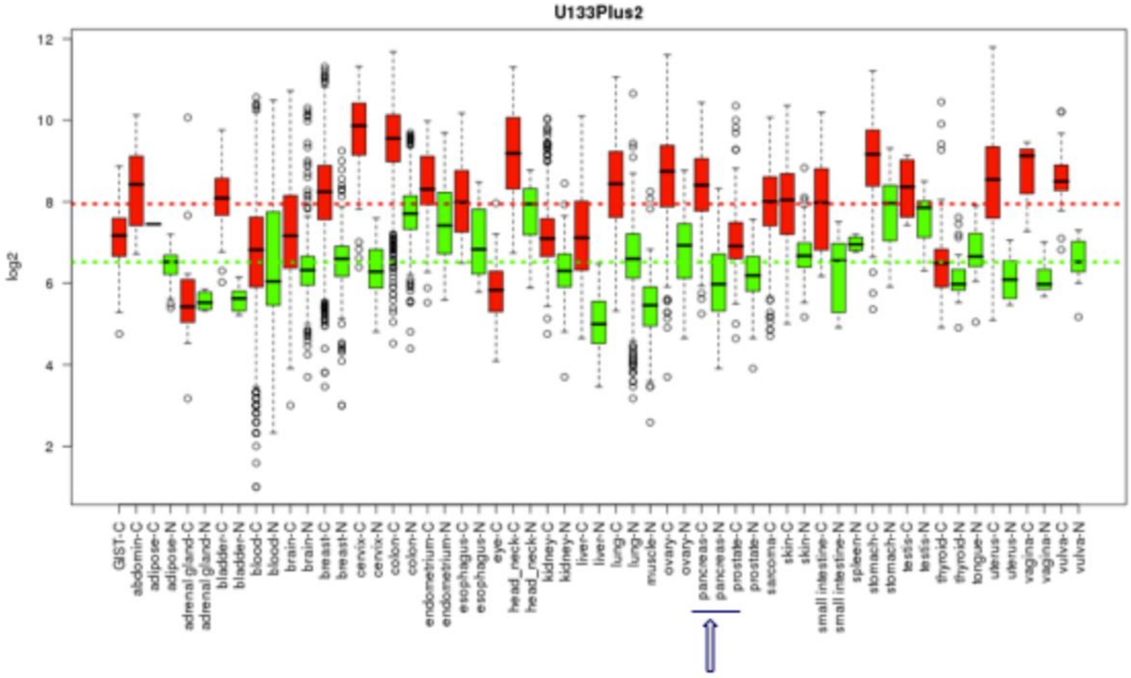
U133Plus2
log2
2
4
6
8
10
12
GIST-C
abdomin-C
adipose-C
adipose-N
adrenal gland-C
adrenal gland-N
bladder-C
bladder-N
blood-C
blood-N
brain-C
brain-N
breast-C
breast-N
cervix-C
cervix-N
colon-C
colon-N
endometrium-C
endometrium-N
esophagus-C
esophagus-N
eye-C
head_neck-C
head_neck-N
kidney-C
kidney-N
liver-C
liver-N
lung-C
lung-N
muscle-N
ovary-C
ovary-N
pancreas-C
pancreas-N
prostate-C
prostate-N
sarcoma-C
skin-C
skin-N
small intestine-C
small intestine-N
spleen-N
stomach-C
stomach-N
testis-C
testis-N
thyroid-C
thyroid-N
tongue-N
uterus-C
uterus-N
vagina-C
vagina-N
vulva-C
vulva-N

TMPRSS4

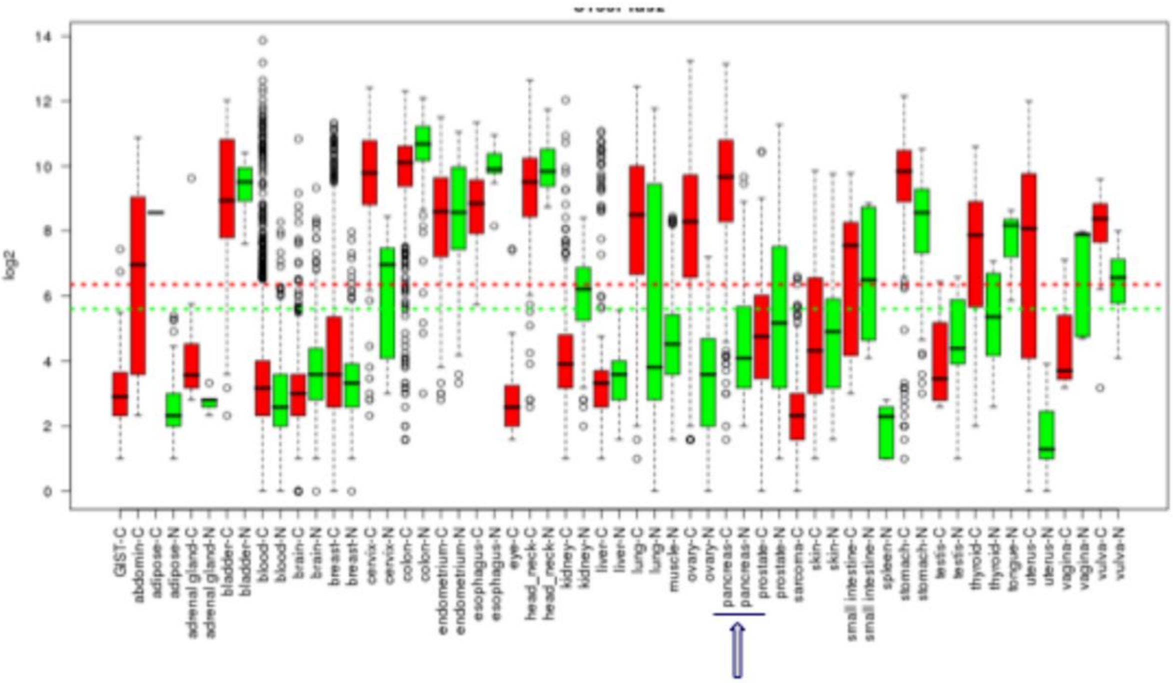

AHNAK2

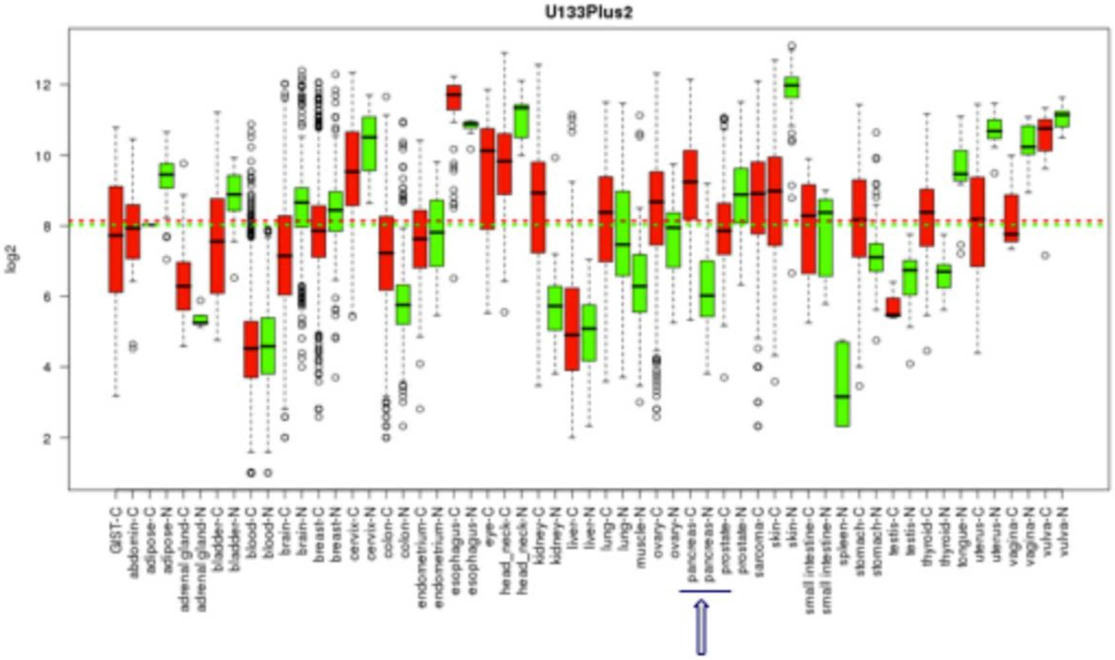

SERPINB5

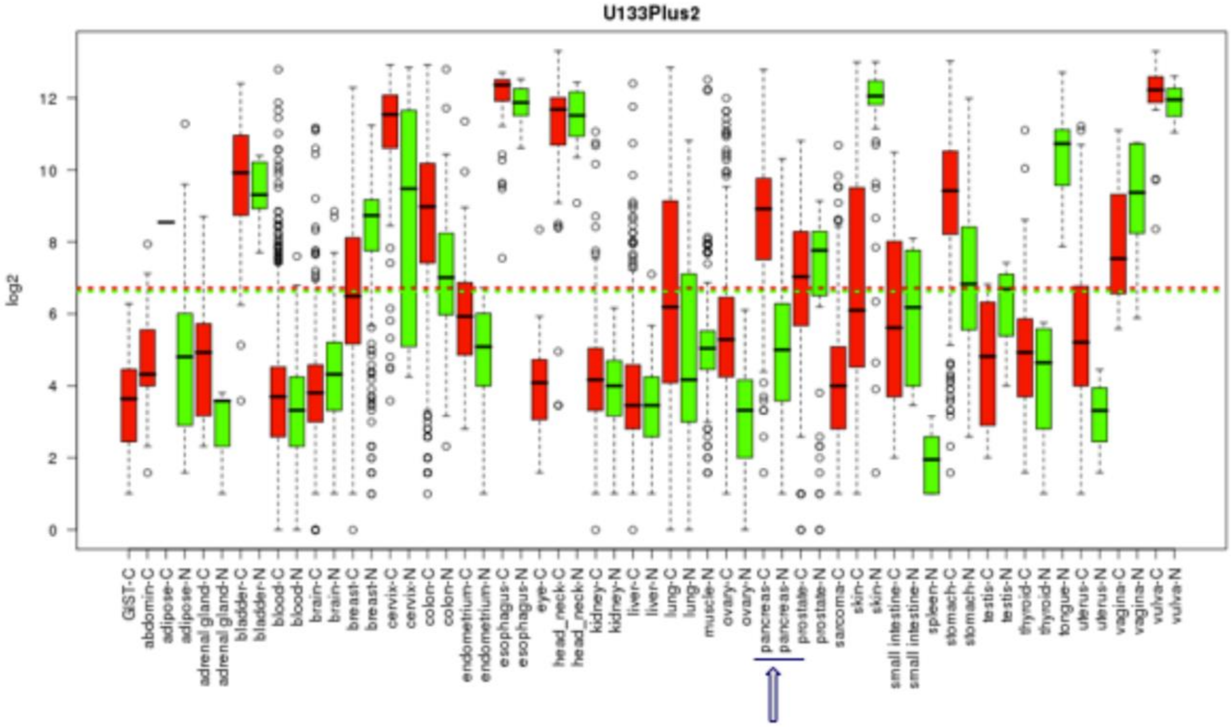

Supplementary Figure S3. GENT database analysis of 5-gene PDAC classifier (TMPRSS4, POSTN, AHNAK2, ECT2, SERPINB5) comparing expression of 5 genes between normal and cancer tissues across different cancer types. TMPRSS4 is overexpressed in cervical, ovarian, gastric, thyroid, and vulvar cancer; SERPINB5 is overexpressed in cervical, colon, ovarian, and gastric cancers; POSTN is overexpressed in brain, breast, esophageal, head and neck, lung, small intestine, thyroid, vaginal, vulvar and testicular cancers; ECT2 is overexpressed in many types of cancer; and AHNAK2 is overexpressed in colon, kidney, stomach, and thyroid cancers.

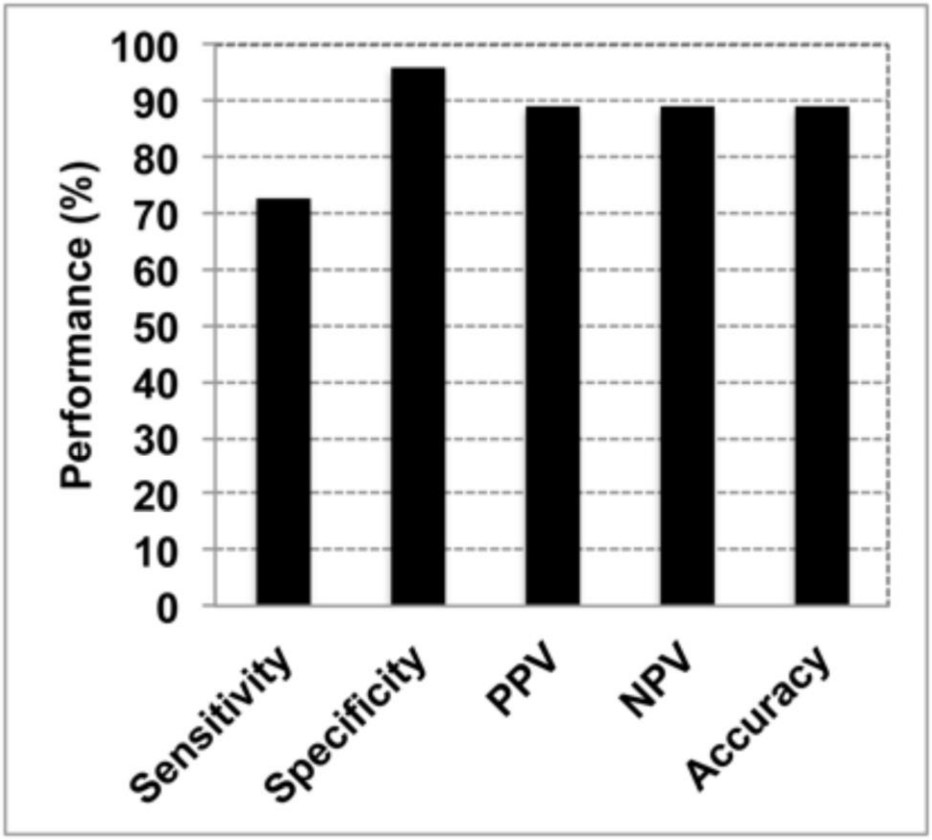
Performance (%)
100
90
80
70
60
50
40
30
20
10
0
Sensitivity
Specificity
PPV
NPV
Accuracy

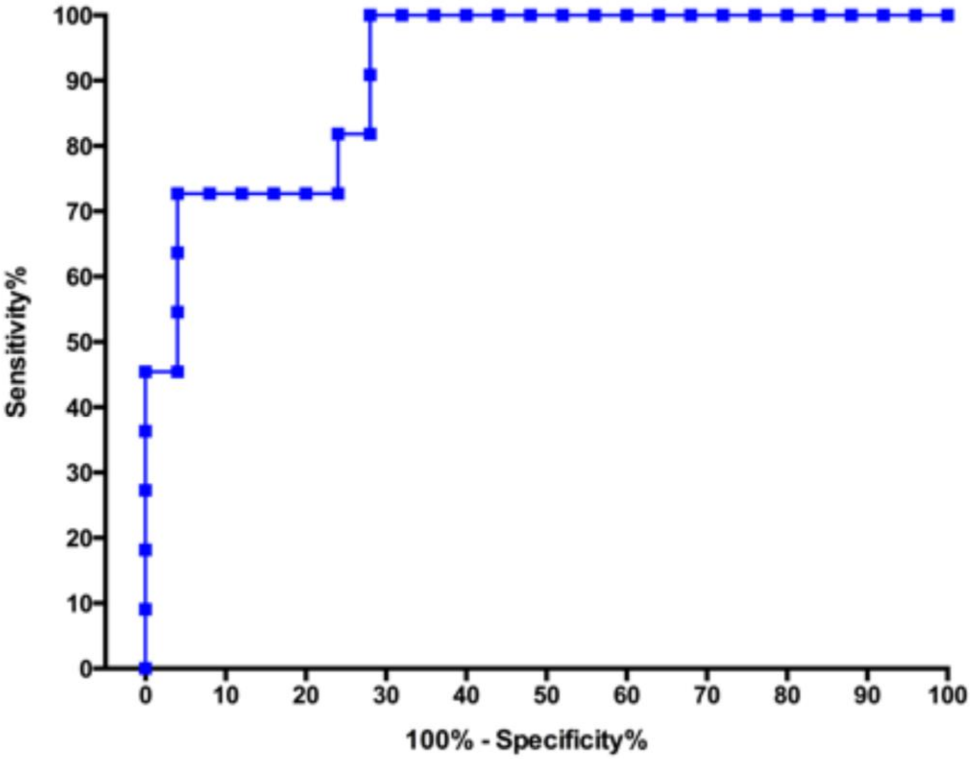
Sensitivity%
100
90
80
70
60
50
40
30
20
10
0
0
10
20
30
40
50
60
70
80
90
100
100% - Specificity%

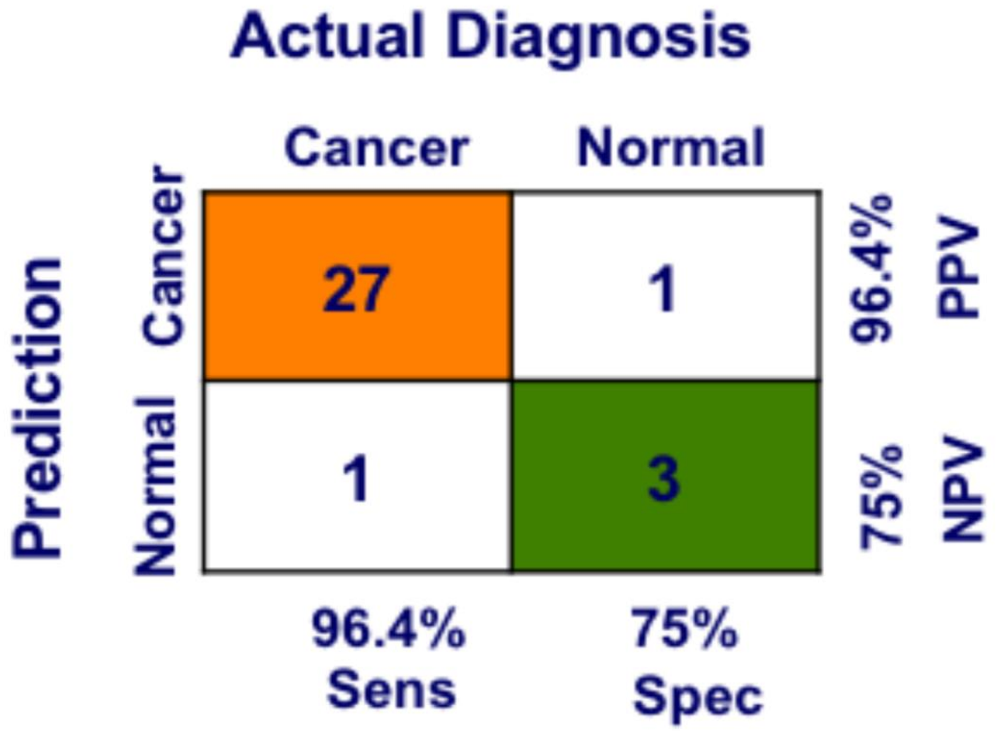

Supplementary Figure S4. PDAC specificity and cross-platform stability analysis.

A) Classification of PDAC vs. Other Cancers (Breast, Colon, Lung). (Top) Diagnostic performance of 5-gene PDAC classifier on a dataset of PDAC, breast cancer, lung cancer and colon cancer tissue samples. PPV = positive predictive value, NPV = negative predictive value. (Bottom) ROC curve for the Multicancer dataset. B) Diagnostic cross-platform performance of 5-gene PDAC classifier for normal vs. PDAC on a dataset using the Agilent platform.

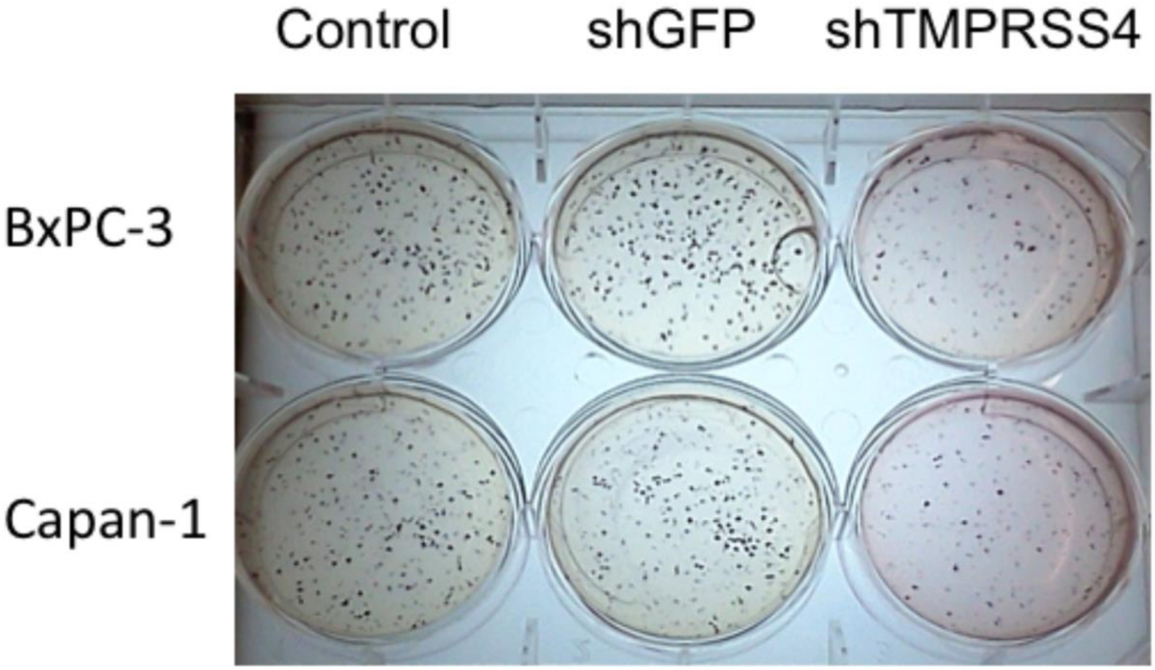

Supplementary Figure S5. Verification of TMPRSS4 function in various PDAC cells.

TMPRSS4 knockdown reduces anchorage-independent growth in Capan-1 and BxPC-3 cells in a soft agar assay. Twenty-four hours after shTMPRSS4 or shGFP infection, Capan-1 and BxPC-3 cells were seeded into soft agar at a density of 1×10^4 per well and allowed to grow for 21 day. Images of stained colonies after 21 day culture are shown.

A)

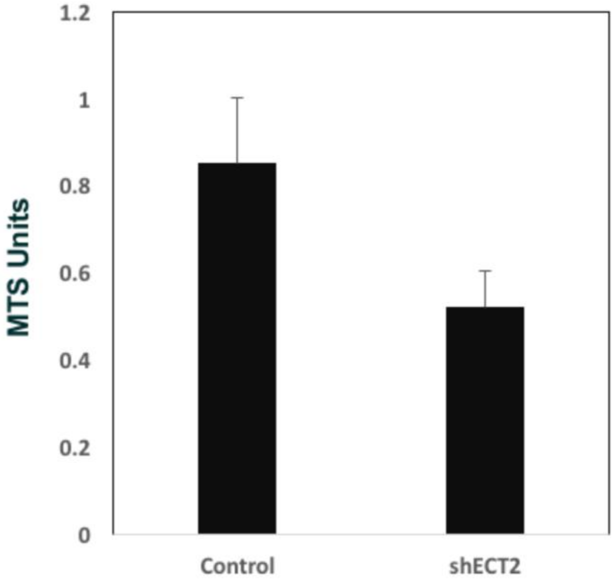
1.2
1
0.8
0.6
0.4
0.2
0
MTS Units
Control
shECT2

B)

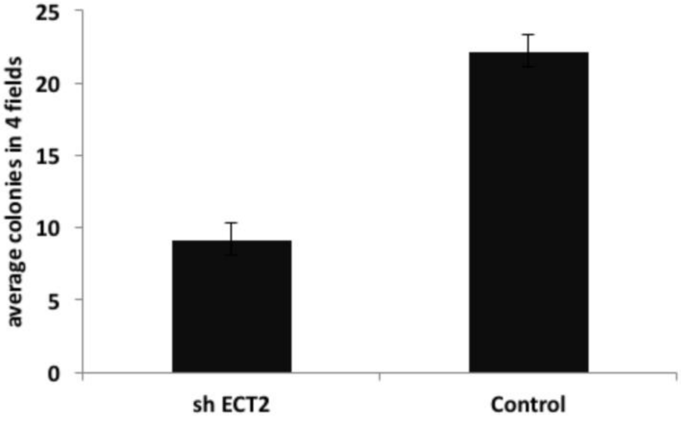
25
20
15
10
5
0
average colonies in 4 fields
sh ECT2
Control

C)

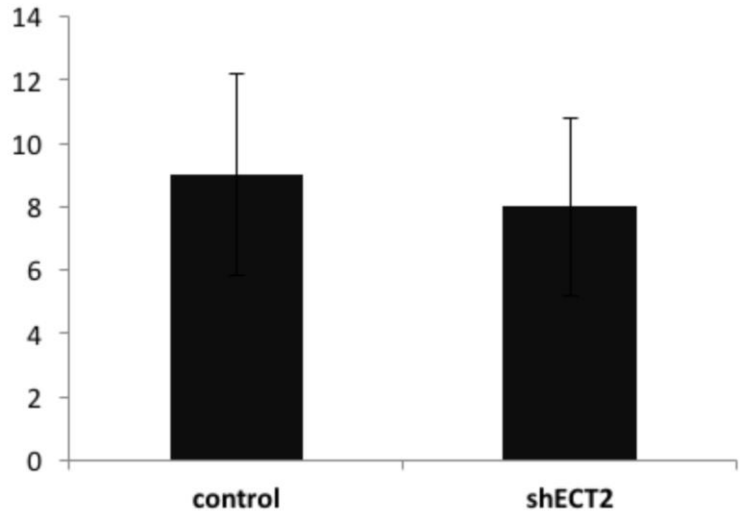

D)

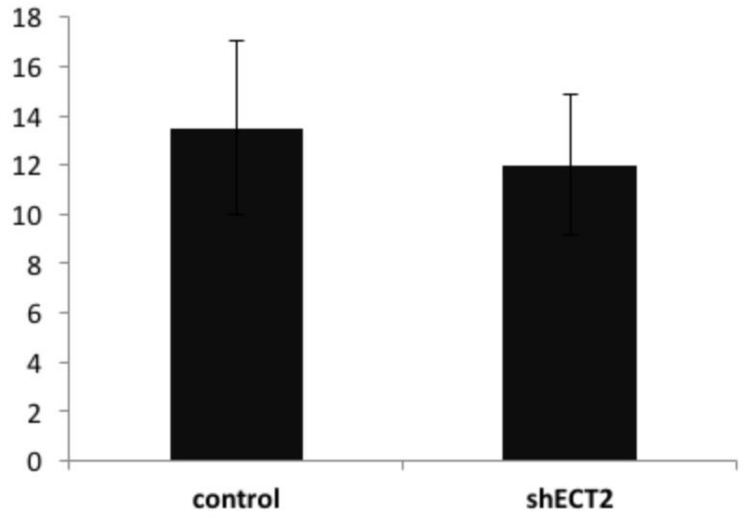

Supplementary Figure S6. Verification of ECT2 function in Capan-1 PDAC cells.

A) Cell viability of ECT2 knockdown cells. Cell viability analysis of shECT2 and scrambled shRNA control cells (Capan-1) using MTS assay. B) ECT2 knockdown reduces anchorage-independent growth in Capan-1 cells in a soft agar assay. Twenty-four hours after shECT2 or scrambled shRNA control infection, Capan-1 cells were seeded into soft agar at a density of 1×10^4 per well and allowed to grow

for 21 day. The average numbers of colonies in 4 field after 21 day culture are shown. C + D) Knockdown of ECt2 reduces migration (C) and invasion (D) of PDAC cells. Capan-1 cells stably transfected with scrambled shRNA-treated cells (control) or ECT2 shRNA (shECT2) were placed in serum-free culture media and added into the upper compartment of a migration or invasion chamber. After 16 hours, cells in the upper chamber were removed and cells that had migrated or invaded through the pores of the membrane to the other side were fixed, stained, and counted. Cells in five different areas were quantified for migration and invasion studies. C, quantification of cells migrating through fibronectin-coated membranes. D, quantification of cells invading through Matrigel-coated membranes after 16-h incubation and 10% serum as chemoattractant.
